# Electrolyzed water for the microbiologic control in the pandemic dental setting: a systematic review

**DOI:** 10.1186/s12903-022-02528-0

**Published:** 2022-12-09

**Authors:** Angélica M. Cárdenas, Vanessa Campos-Bijit, Fabrizio Di Francesco, Frank Schwarz, Emilio A. Cafferata, Rolando Vernal

**Affiliations:** 1 Periodontal Biology Laboratory, Faculty of Dentistry, Universidad de Chile, Sergio Livingstone Pohlhammer 943, 8380492 Santiago, Independencia Chile; 2 Faculty of Dentistry, Universidad Santo Tomás, Bucaramanga, Colombia; 3 Department of Science and Innovation, BIOMEP Research Group, Bucaramanga, Colombia; 4 Multidisciplinary Department of Medical, Surgical and Dental Sciences, Campania University Luigi Vanvitelli, Naples, Italy; 5 Department of Oral Surgery and Implantology, Carolinum, Johann Wolfgang Goethe-University Frankfurt, Frankfurt, Germany; 6 Department of Periodontology, School of Dentistry, Universidad Científica del Sur, Av. Paseo de la República 5544, 15074 Lima, Miraflores Peru

**Keywords:** Electrolyzed water, COVID-19, Microbiology, Dental setting, Systematic review

## Abstract

**Background:**

Electrolyzed water has brought recent attention due to its antimicrobial properties. Indeed, electrolyzed water has been proposed to sterilize dental materials and instruments without compromising their structural integrity. In addition, electrolyzed water has been proposed as a mouthwash to control bacterial and viral oral infections without detrimental effects on the oral mucosa. However, no current consensus or evidence synthesis could indicate its potentially favorable use in the dental setting, particularly during the COVID-19 context. Therefore, this systematic review aimed to elucidate whether electrolyzed water could improve microbiologic control in the COVID-19 pandemic dental setting.

**Methods:**

MEDLINE via Pubmed, EMBASE, Cochrane’s CENTRAL, Scopus, LILACS, and Web of Science databases were searched up to September 2021 to identify experimental studies utilizing electrolyzed water for eliminating microorganisms in a dental setting. Besides, a manual and a grey literature search were performed. The data selection and extraction were performed individually and in duplicate. The Risk of Bias (RoB) was assessed with the Nature Publication Quality Improvement Project (NPQIP) score sheet. The study protocol was registered at PROSPERO CRD42020206986.

**Results:**

From a total of 299 articles, 63 studies met the inclusion criteria. The included studies assessed several types of electrolyzed waters, which showed a high disinfection potential when used to deal with different oral conditions. Electrolyzed water demonstrated a broad antimicrobial spectrum and was highly efficient in the dental office disinfection against viruses, fungi, and bacteria, being compatible with most dental materials. In addition, electrolyzed water could protect against SARS-CoV-2 infection and contamination in the dental office. Regarding the RoB, only 35.18% of entries were answered as ‘Yes’, thus achieving less than half of the reporting sheet.

**Conclusion:**

Electrolyzed water effectively disinfects contaminated surfaces, dental materials, and equipment. Therefore, their use is recommendable in the SARS-CoV-2 pandemic dental setting.

**Supplementary Information:**

The online version contains supplementary material available at 10.1186/s12903-022-02528-0.

## Background

The worldwide impact of coronavirus disease 2019 (COVID-19) is affecting a still increasing number of people daily. The uncontrolled widespread of severe acute respiratory syndrome coronavirus 2 (SARS-CoV-2) infection led to the global expansion of COVID-19 in over 200 countries, with stunning contagion and mortality rates [1]. Recently, the coronavirus’ genomic mutations and resulting variants have increased its virulence and infective potential, facilitating zoonotic and human transmission [2, 3].

The main transmission route of the virus is via the release of saliva droplets or aerosols by COVID-19-infected people by coughing, sneezing, talking, or touching contaminated surfaces without adequately washing hands. Accordingly, significant loads of SARS-CoV-2 RNA have been detected in saliva [4], salivary glands [5], and oral epithelial cells [6]. Indeed, the SARS-CoV-2 virus can colonize the oral mucosa by the differential expression of angiotensin-converting enzyme II (ACE2), the SARS-CoV-2 main receptor and cell-invasion route [6]. Therefore, considering the importance of the oral cavity as the primary source of viral transmission of COVID-19, the conditions of clinical dental care should be a focus of attention. Certainly, the length of dental procedures and the proximity between patients and operators within the dental setting put dentists and their patients at high risk of COVID-19 [7]. Moreover, the constant use of water-cooled high-speed rotary hand-pieces or ultrasonic scalers during most dental procedures generates and further spreads virion-loaded aerosols [8, 9]. Indeed, these aerosols can contaminate surfaces, dental instruments, impression casts, and dental unit-waterlines, among others, increasing the risk for cross-contamination [8, 9].

Several disinfectants and cleaning solutions are currently available for microbiologic control at the dental office, including viruses. However, most of them are not entirely innocuous for humans, dental instruments, materials, and the environment and are not fully effective in controlling the virus dissemination [10, 11]. From this basis, the use of electrolyzed water has brought recent attention due to its bactericidal and virucidal activities and less detrimental effects on biological tissues, skin, and mucosa [12]. Its antimicrobial properties have allowed it to sterilize instruments, dentures, and even impressions, without compromising their delicate structural integrity [13]. Furthermore, it has been hypothesized that its use as a mouthwash could be innocuous for the oral mucosa [14]. Thus, it could effectively reduce the SARS-CoV-2 viral load in patients’ saliva before aerosol-generating procedures. However, nowadays, there is no current consensus or evidence synthesis that could indicate its potentially favorable use in the dental setting. Therefore, this systematic review aimed to elucidate whether electrolyzed water could improve the microbiologic control in the COVID-19 pandemic dental setting.

## Methods

### Study protocol

The protocol for this systematic review was constructed in agreement with the recommendations made by the PRISMA 2020 checklist [15] and following the quality standards recommended by the AMSTAR-2 critical appraisal tool [16]. All the authors revised, discussed, and approved the protocol for the data selection and extraction, risk of bias assessment, and data analysis a priori. Protocol registration can be found at PROSPERO CRD42020206986.

### Eligibility criteria

To answer the research question: Does electrolyzed water improve microbiologic control in the dental setting? Publications that met the following PICO format and inclusion criteria were included.

- P: Oral microorganisms including viruses, bacteria, and fungi cultivated in vitro, inoculated in an animal model, or sampled from the oral cavity.
- I: Electrolyzed water produced by electrolysis of regular or distilled water with any concentration of sodium chloride.
- C: No intervention or other disinfectant solution used in the dental setting.
- O: Colonies count, plaque reduction, infection potential, and viral RNA copies.

Due to the relative novelty of the revised topic in the dental setting, randomized clinical trials were scarce. Thus, all experimental studies were considered, including randomized or non-randomized controlled trials, animal studies with a control group, and in vitro studies. Case reports, editorial letters, and incomplete studies were excluded. Language, year, and publication status were not considered as exclusion criteria. The search retrieved articles published up to September 2021, and eligible publications in languages different from English, Spanish, French, or Portuguese were translated.

### Information sources and search strategy

One author (EAC) performed the electronic search in MEDLINE via Pubmed, EMBASE, Scopus, Web of Science, Cochrane’s CENTRAL, and LILACS databases, using individually adapted search strategies (Supplementary Material 1). In addition, OpenGrey and PQDT-ProQuest databases were searched for grey literature. Moreover, a manual search including recently published records from 2021 was performed in the journals from which initial studies were selected: *International Journal of Oral Biology*, *International Journal of Clinical Preventive Dentistry*, *Journal of the Korean Academy of Pediatric Dentistry*, *Journal of Dental Rehabilitation and Applied Science*, *Oral Health and Dental Management*, *Tropical Journal of Pharmaceutical Research*, *Annals of Pathology and Laboratory Medicine*, *International Journal of Applied Pharmaceutics*, *Brazilian Dental Journal*, *International Journal of Applied Dental Sciences*, *Journal of Oral Research and Review*, *Biomedical Research*, *Journal of Oral Science*, *The Journal of the Japanese Society for Dental Materials and Devices*, *BioMed Research International*, *Jundishapur Journal of Microbiology*, *Dental Materials Journal*, *Journal of the Japanese Prosthodontics Society*, *The Japanese Journal of Conservative Dentistry*, *The Journal of the Kyushu Dental Society*, *Japanese Journal of Oral Biology*, *Journal of Dental Health*, *Journal of Clinical Periodontology*, *Journal of Virological Methods*, *Japanese Journal of Dental Materials*, and *Journal of Microbiology*. Besides, contact with corresponding authors was made via e-mail if some potentially eligible detected article was unavailable. Finally, the references of the retrieved studies were also revised for additional studies.

### Data selection and extraction

Two authors (AC and VC) performed the data selection and extraction independently. After duplicate removal, titles and abstracts were assessed for their potential inclusion. Then, full-text articles were analyzed against the inclusion criteria for their final inclusion. When disagreements occurred, inclusion was discussed with a third author (RV) until consensus. Excluded articles and their respective reasons for exclusion were recorded (Supplementary Material 2). Afterward, the following data were extracted from the included studies: Authors, year, and study setting (for reference), type of study, microorganism (source, concentration, and inoculation via), type of hydrolyzed water (concentration and preparation), duration of intervention, dental setting and comparator, effect over microorganism (time frame), and funding source, if available. Disagreements were solved by discussion, and all the extracted data were further revised by two authors (EAC and RV) to ensure the extraction of all the relevant data. In the case of missing data, the manuscripts’ corresponding authors were asked for additional information.

### Outcome measures

In order to evaluate the potential use of electrolyzed water for microbiologic control in the dental setting, the primary outcome measure was the inhibition of bacterial growth by means of remaining colony forming units (CFU) or viral replication by means of remaining viral RNA copies.

### Risk of bias assessment

The risk of bias (RoB) was assessed independently and in duplicate by two reviewers (AC and EAC), previously calibrated in RoB assessment rounds, and the disagreements were resolved by consensus. The in vivo and in vitro studies were assessed using a modified version of the Nature Publication Quality Improvement Project (NPQIP) score sheet.

### Data synthesis

Due to the substantial heterogeneity between studies, a qualitative synthesis of results was performed.

## Results

### Data selection

**Fig. 1:**
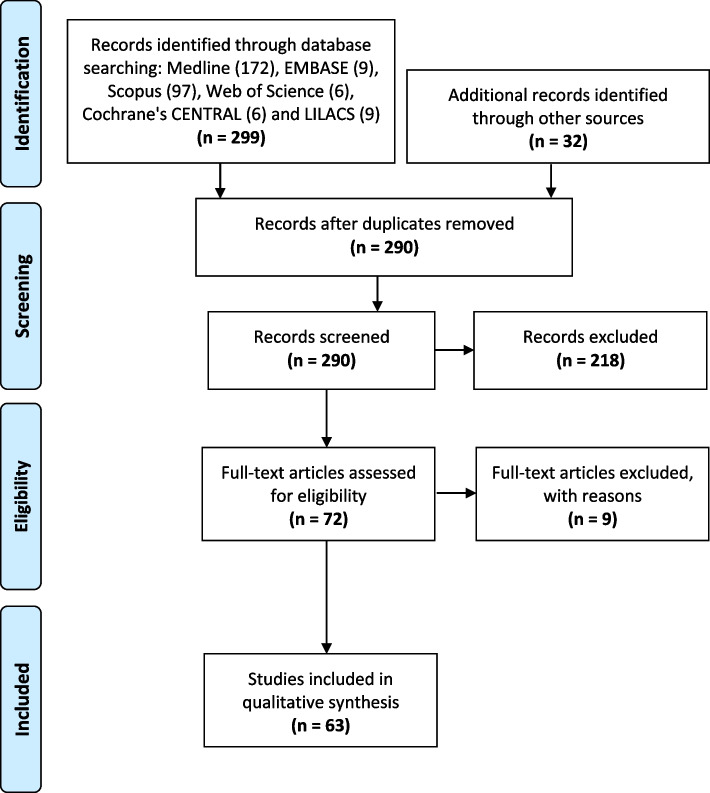
PRISMA Flow chart of data selection process

The initial electronic search yielded 299 articles across the revised databases. Additionally, the complementary manual search resulted in 32 additional records. After duplicates were removed, 290 articles were screened. Then, revising titles and abstracts excluded 218 records, resulting in the assessment of 72 potential articles, including nine articles translated from Japanese, Korean, and Turkish. Finally, the full-text assessment resulted in the inclusion of 63 studies (Fig. 1). The main reasons for excluding full-text articles were: not being primary studies, using another disinfectant different from electrolyzed water, being incomplete, not being executed in the dental setting, and not evaluating oral microorganisms. All the included studies were in vitro except for five studies [17–21] which were randomized clinical trials.

### Disinfection potential of electrolyzed water for oral diseases

Different varieties of electrolyzed water have been proven to be potentially helpful in treating oral diseases. In the case of caries, patients rinsing with ‘electrolyzed hydrogen water’ showed significantly diminished counts of *Streptococci* CFU in comparison with rinsing with tap water, leading to the authors’ concluding that oral rinse with electrolyzed water could be supportive in diminishing the levels of caries-associated bacteria such as *Streptococcus mutants* [17]. Otherwise, in periodontal diseases, electrolyzed water has been tested in three randomized clinical trials. For instance, in periodontally healthy individuals, irrigation with ‘aqua oxidizing water’ after discontinuing oral hygiene was effective for plaque control and preventing gingivitis [19]. Likewise, the use of ‘acid water’ rinse inhibited the development and formation of bacterial plaque, similar to chlorhexidine in human dentine [21]. Apart from that, ‘superoxidized water’ irrigation along with scaling and root planning was effective for treating periodontitis-affected patients, showing diminished probing pocket depth and gingival bleeding 30 days after treatment [20].

Apart from that, the canal-disinfection potential of electrolyzed water has particularly been considered in endodontics. Indeed, a randomized clinical trial showed no differences between the use of ‘oxidative potential water’ in comparison with 1% NaOCl, in terms of their effectiveness in reducing the bacterial load in canals of necrotic pulpectomized primary teeth [18]. In particular, the antimicrobial properties of electrolyzed water against *Enterococcus faecalis* in canals of extracted teeth have been tested in seven studies [20, 22–27], being effective in reducing *E. faecalis* levels as an endodontic irrigant, in a similar manner to sodium hypochlorite solution (Table 1).

**Table 1:** Electrolyzed water used in the human dental setting

	Publication	Type of study	Objective	Subject/Population	Intervention	Comparison	Outcome measures	Main results	Authors’ conclusions
1	Kim J., et al. 2017 [17]	RCT	To evaluate the effectiveness of electrolyzed hydrogen water against oral *Streptococci* biolfims.	6 healthy adults with at least 20 teeth.	Oral rinse with electrolyzed Hydrogen water (H-water): dissolved hydrogen, 1.5 ppm; oxidation-reduction potential, − 600 mV ~ − 700 mV. Patients rinsed 3 times dayly with the test intervention for 2 weeks, then after pausing for 1 week, proceeded with tap water oral rinses for two weeks.	Tap water oral rinse	Mean ± SD of *Streptococci* CFU in saliva	After one week, four of the six participants showed significantly lower streptococcal CFU after gargling with H-water than after gargling with tap water (*p < 0.05, **p < 0.01). For all 18 trials data, The CFU values were also significantly lower when oral rinse was performed with H-water (*p* < 0.005).	Oral rinse with electrolyzed water would be helpful in diminishing oral *Streptococci* related to caries.
2	Valdez-Gonzales C., et al. 2013 [18]	RCT	To evaluate the effectiveness of OPW in reducing bacterial loading as an irrigating solution in necrotic pulpectomized primary teeth.	40 child patients between 3 to 8 years with at least 1 necrotic teeth needing canal treatment.	Canal irrigation with oxidative potential water (OPW):+ 1100 mV, pH and ORP greater than 2.7.	Canal irrigation with 1% NaOCl	Number of CFU/mL on teeth with canal before and after irrigation.	In the pre-irrigation samples corresponding to the OPW group, a median of 1.65 × 10^9^ CFU/mL (range, 9 × 10^8^–2.1 × 10^9^) with a mean of 1.63 × 10^9^ ± 4.18 × 10^8^ CFU/mL was obtained. In the same experimental group for post-irrigation samples, a median of 0 CFU/mL (range 0–5 × 10^8^), with a mean of 5 × 10^7^ ± 1.53 × 10^8^ CFU/mL, was obtained. The difference between the bacterial load after irrigation with OPW was statistically significant (*P* < 0.0001). Comparative analysis showed no statistically significant difference between 1% NaOCl and OPW (*P* = 0.1519).	Electrolyzed water could be a feasible alternative for irrigating after pulpectomy of necrotic primary teeth.
3	Gulabivala K., et al. 2004 [22]	In vitro	To test the effectiveness of electrochemically activated aqueous solutions in the debridement of *E. faecalis* biofilms in root canals of extracted teeth.	198 extracted human single-rooted teeth.	Canal irrigation with electrochemically activated water: Neutral anolyte (NA) (pH 6.5), acidic anolyte (AA) (pH 3.0), or catholyte (C) (pH 11.5).	Solutions with and without ultrasonication. PBS, NaOCl (3%)	Remaining CFU/mL	The NA, NA (U), C alternated with NA and the AA (U) groups all had significantly (α = 0.05) lower CFU counts compared with PBS controls.	There was a significant difference between the C/NA groups with and without ultrasonication but not between other combinations. NA (U) and AA (U) were the most effective test solutions but NaOCl (3%) gave by far the highest bacterial kills.
4	Marais JT, Williams WP. 2001 [23]	In vitro	To evaluate the antimicrobial effectiveness of ECA on a selected group of anaerobic bacteria in root canals of extracted human teeth.	60 caries-free, single-rooted, adult maxillary anterior human teeth were collected from the extraction clinic, intracanal Irrigated for 5 min with one of the four different irrigation solutions.	Electro-chemically activated water (ECA): pH 7.0 or pH 9.0.	Distilled water, Sodium hypochlorite (3.5% concentration).	Mean (SD) of CFU/mL of *P. intermedia, P. gingivalis, E. faecalis,* and *A. actinomycetemcomitans.*	ECA groups showed fewer numbers of colony formation 693 (253), 525 (418) respectively. This reduction however, did not approach the negative (zero) values as recorded for NaOCl and in fact are closer to those values obtained for the control group (group A).	The use of ECA caused a reduction in the number of anaerobic bacteria within the root canal system, but this was not statistically significant (*P* > 0.05) when compared to sodium hypochlorite.
5	Zan R., et al. 2016 [24]	In vitro	To evaluate and compare the antibacterial efficacy of SPO on *E. faecalis* biofilms in human root canals at different irrigation times.	126 extracted human mandibular premolar teeth with a single canal.	Canal irrigation with super-oxidized water (SPO) for different periods of time.	Canal irrigation with 0.9% NaCL, 5.25% NaOCl for 2 minutes.	CFU/mL *E. faecalis.*	There were statistically significant differences between negative control and all groups (*P* < 0.05). Although positive control showed no statistically significant difference when compared with SPO groups.	Super-oxidized water had a highly antibacterial effect against *E. faecalis* biofilms in root canals. Moreover, super-oxidized water indicated a remarkable and similar bactericidal effect to that of traditional NaOCl against *E. faecalis* biofilms.
6	Hope CK., et al. 2010 [25]	In vitro	To elucidate the importance of the biofilm modality of growth of *E. faecalis* with respect to its recalcitrance during endodontic irrigation.	8 extracted human single teeth.	Super-oxidised water (SOXH2O), pH 5–6.5, components: Sodium chloride (0.42%w/v), hypochlorous acid (0.022%w/v) and sodium chlorate (0.002%w/v).	1% NaOCl and PBS.	Mean log CFU/mL of *E. faecalis.*	Remaining mean log CFU of *E.faecalis* from SOXH2 irrigated teeth was greater than 1% NaOCl (4.66 vs 3.36), without statistical significance (*P* = 0.177).	Biofilms of *E. faecalis* were susceptible to concentrations of irrigant that proved ineffective in the tooth model.
7	Ezure M., et al. 1996 [translated from Japanese] [19]	RCT	To evaluate the effect of oral hygiene with Water-spray-type Oral Washing Unit and Aqua Oxidizing Water on experimental gingivitis.	15 males with clinically healthy gingiva discontinuing or not oral cleaning.	Oral irrigation with aqua oxidizing water (AOW) three times a day during 30 s.	Discontinuation of all oral cleaning, and oral washing with sterile distilled water.	The change of the plaque index (P1I), the gingival index (GI), and gingival crevicular fluid (GCF volume), bacteria flora, halitosis for 1 and 2 weeks.	Changes in P1I showed significant reduction in the negative control group and test group in comparision with the controls (p < 0.05, p < 0.01). The time course of changes in GCF volume showed significant changes compared to the negative control group and the test group(*p* < 0.05, *p* < 0.01). Based on the results above, a plaque-controlling effect due to the use of the AOW was recognized, and an effect on bacterial flora due to use of AOW as the oral washing agent was observed.	The use of aqua oxidizing water for irrigation is useful in exerting a plaque-controlling effect and a gingivitis-preventing effect.
8	Ruqshan Anjum MG., et al. 2015 [26]	In vitro	To determine the antimicrobial efficacy of chlorhexidine, Oxum, Ozonated water in root canals infected by *E. faecalis.*	40 extracted single-rooted human teeth.	Canal irrigation with super oxidized water, (Neutral pH), components: Hypochlorous acid, Sodium hypochlorite, Chlorine dioxide, Ozone, Hydrogen peroxide, and Sodium chloride.	2% chlorhexidine, Ozonated water and NaCl.	CFU/mL of *E. faecalis.*	CFU of *E. faecalis* decreased in the pre-test when treated with CHX followed by super-oxidized water, ozonated water and saline.	Chlorhexidine significantly reduced the number of *E. faecalis* followed by super-oxidized water and ozonated water.
9	Chaudhari H., et al. 2019 [20]	RCT	To compare the superoxide solution with povidone-iodine by means of clinical parameters and microbiologically by CFU.	20 sites with chronic periodontitis (PD ≥ 5 mm) (ten sites per group). All patients received SRP.	Superoxide solution.	Povidone-iodine.	Mean/SD of Probing pocket depth (PPD) and sulcus bleeding index (SBI). CFU/mL.	The mean PPD at baseline and 30 days was observed to be 1.716 ± 0.351 and 0.683 ± 0.274, respectively, for EWA and 1.700 ± 0.380 and 1.0 ± 0.00, respectively, for control. The mean gingival sulcus bleeding scores at baseline and 30 days were observed to be 1.726 ± 0.351 and 0.603 ± 0.274, respectively, for EW and 1.700 ± 0.380 and 1.25 ± 0.00, respectively, for control. There was a statistically significant reduction in CFU in EW after 1 month as compared to control.	Superoxidized water irrigation as an adjunct to SRP proved to be effective in the treatment of periodontitis, without any side effects.
10	Ito K., et al.; 1996 [21]	RCT	To compare the effects of Acid Water with placebo treatment on the ultrastructure of early plaque formed on dentine specimens attached to retainers in the human oral cavity.	24 dentine specimens from 12 freshly extracted healthy human teeth placed in retainers. The retainers were placed in 6 healthy participants.	Acid water (AW): pH < 2.7, ORP > 1100 mV, active oxygen and Chlorine.	2% chlorhexidine, saline.	Morphology and developmental condition of plaque deposits. Thickness of plaque formed in mm.	On AW treatment, the plaque consist of coccoid forms and and short rods, the plaque is not well developed. The thickness of plaque accumulation was moderate (8.80 mm). On CHX treatment, plaque developed was fairly and was composed mainly cocci, very few short rods and filaments were hardly evident. There wasn’t statistically significant difference between AW and CHX (p < 0.001).	Washing with AW has almost the same potential for inhibition of plaque formation as washing with CHX without producing any side-effects. Therefore, there is a possible use for AW as an anti-plaque.
11	Lata S., et al. 2016 [27]	In vitro	To compare and evaluate the antimicrobial effectiveness of ECA, 1% hypochlorite, and 3% hypochlorite when tested against the standard strain of *E. faecalis*.	48 extracted human permanent maxillary central incisors of patients between the age group 40 to 60 years irrigated with different solutions for 5 minutes.	Electro-chemically Activated (ECA) water.	1% an 3% sodium hypochlorite, distilled water.	Mean (μL)/SD CFU and Mean (nm)/SD CFU of *E. faecalis,* before and after irrigation.	Differences between CFU values between groups was found to be not statistically significant. There was a statistically significant difference between the optical density values among sodium hypochlorite, and EW.	The antibacterial efficacy of ECA water was found to be comparable with sodium hypochlorite solution against *E. faecalis*.
12	Hope CK., et al. 2010 [25]	In vitro	To validate an extracted tooth model of endodontic irrigation.	Twelve extracted, single rooted teeth.	Super-oxidised water (SOXH_2_O).	PBS, 1% NaOCl, 2% CHX.	The number of viable bacteria recovered, mean (Log_10_ CFU) *E. faecalis.*	The number of viable bacteria recovered from the teeth following irrigation with the PBS control was 3.329 (log_10_ cfu), whilst the antimicrobial irrigants 1% NaOCl, 2% CHX and SOXH2O yielded 0.552, 1.441 and 1.577 (log_10_ cfu) respectively. However, only the difference between PBS and 1% NaOCl was statistically significant.	The extracted tooth model is a useful method for evaluating the effectiveness of antimicrobial endodontic irrigants. In these preliminary experiments, the most effective irrigant was 1% NaOCl.

### Innocuity of electrolyzed water in vitro and for human application

The innocuity of electrolyzed water has been tested using in vitro models and randomized clinical trials [17–21]. Indeed, daily oral rinse, irrigation, or application of ‘electrolyzed water’ reported no adverse effects on patients [17–21], while having an outstanding plaque-controlling effect. Besides, canal irrigation of necrotic primary teeth on child patients with ‘oxidative potential water’ reported no side effects, thus considering it a harmless alternative for irrigation after pulpectomies [18]. Moreover, electrolyzed water was not cytotoxic to cultured human bone marrow-derived mesenchymal stem cells [28].

### Oral microorganisms growth inhibition by electrolyzed waters

The antibacterial properties of electrolyzed water were tested in 32 in vitro studies [17, 22, 29–58] (Table 2). In the case of caries-associated bacteria, the exposition to electrolyzed water was effective in diminishing bacteria CFU counts, inhibiting colonies or biofilm formation, mostly by *S. mutants* and, in some cases, *S. sobrinus*, *S. mitis*, *S. sanguis*, and *S. salivarius* [29, 38, 43, 45, 50, 51, 54, 58]. Moreover, the inoculation of electrolyzed water was able to inhibit periodontopathogens such as *Porphyromonas gingivalis*, *Aggregatibacter actinomycetemcomitans*, *Fusobacterium nucleatum*, *Prevotella intermedia*, and *Treponema denticola* [22, 29, 32, 35, 38, 41, 42, 45, 49, 50, 54, 56], by significantly reducing the percentage of viable bacteria or their CFU number after treatment. In addition, pathogens commonly found on infected canals, such as *E. faecalis* and *Porphyromonas endodontalis*, were also sensitive to electrolyzed water, showing significantly reduced CFU numbers and viability in a time-dependent manner [29, 31, 37, 39, 43, 52, 55]. Additionally, electrolyzed water also showed antifungal activity by inhibiting the growth and diminishing the mean CFU counts of *Candida albicans* cultures in nine studies [29, 31, 34–36, 47, 52, 59, 60] (Table 3). Last but not least, electrolyzed water showed viricidal effects against human hepatitis B virus, human immunodeficiency virus, poliovirus type 1, and herpes simplex virus type 1, by reducing their infectivity potential in a time-dependent manner [47, 61, 62] (Table 4).

**Table 2:** Electrolyzed water used against cultured oral bacteria

	Publication	Setting	Type of study	Type of question	Subject/Population	Type of water	Manufacture	Comparison	Dependant variable(s)	Main results	Authors’ conclusions
1	Okamura T., et al. 2019 [29]	Nihon University, Tokyo, Japan	In vitro	To assess antimicrobial and noxious effects of acid/alkaline electrolyzed FW compared with NaOCl	*S. mutans, E. faecalis, C. albicans,* and *P. gingivalis.*	Acid electrolyzed functional water. Acid: ACC 30 ppm; pH 2.7; ORP > 1100 mV) and alkaline: ACC 0 ppm; pH 11.5; ORP ≈ 800 mV	FW were provided by Miura Denshi (Nikaho, Japan).	NaOCl 6%.	Mean ± SD CFU/mL and Mean ± SD viable cell number.	Colony numbers of *S. mutans, P. gingivalis,* and *E. faecalis* were significantly reduced after treatment with acid FW. Alkaline FW showed strong bactericidal effects only for *P. gingivalis*. Further treatment for longer periods yielded a time-dependent decrease in viability; no colony was present after 20 min of treatment.	Acid FW is safe and has a bactericidal effect equivalent to that of NaOCl. Because of its efficient bactericidal, and less noxious, effects on human cells, acid FW may be a useful irrigant for effective root canal treatment.
2	Kim J., et al.; 2017 [17]	School of Dentistry, Kyungpook National University, Daegu. South Korea	1. In vitro 2. RCT	To evaluate the effect of H-water on oral *Streptococci.*	*S. mutans* and *S. sobrinus*	Electrolyzed Hydrogen water (H-water): dissolved hydrogen, 1.5 ppm; ORP, −600 mV ~ −700 mV.	Nanotec (nano H®, South Korea)	Tap water.	Absorbance at 590 nm.	Bacteria incubated with H-water did not form any colonies.	Oral rinse with H-water would be helpful in treating dental biofilm-dependent diseases with ease and efficiency.
3	Gunaydin M., et al.; 2014 [31]	Ondokuzmayis University, Turkey	In vitro	To investigate the in-vitro activity of superoxidized water against an extended group of microorganisms including bacteria and fungi causing hospital-acquired infections.	Six ATCC strains: *A. baumannii, E. coli, E. faecalis, K. pneumoniae, P. aeruginosa, S. aureus,* eight multidrug-resistant bacteria isolated from different clinical samples: *A. baumannii, E. coli, vancomycin resistant E. faecium, K. pneumoniae, P. aeruginosa, methicillin-resistant S. aureus, B. subtilis,* and *Myroides* spp.	Super-oxidized water: pH 6, 80 ppm chlorine	Medilox® (Soosan E & C, Korea) device uses salt, water and electricity and electrolyzes water.	Different concentrations and contact times of SOW.	Absence/presence of growth of ATCC strain, multidrug-resistant bacteria.	Medilox® was effective against all standard strains and all clinical isolates tested at a dilution of 1/1 and exposure time of 1 minute.	SOW produced by Medilox® disinfectant generator using water, salt, and electricity provides highly efficient disinfection.
4	Okajima M., et al. 2011 [32]	Meiji Pharmaceutical University, Tokyo, Japan	In vitro	To evaluate the bactericidal action of ERI on periodontopathic bacteria.	*P.gingivalis, A. actinomycetemcomitans*	Electrolyzed ion-reduced water (ERI): pH of 12.0–12.4, ORP − 344 mV, corresponding to a 0.3% NaCl solution	ERI generator (A. I. System Product Corp.)	ERI solution containing 1% Sodium carboxymethyl cellulose (CMC-Na), 0.3% NaCl solution.	CFU/mL; viable cell count of *P. gingivalis* and *A. actinomycetemcomitans.*	More than 99 and 100% of each bacteria species were killed after exposure to ERI or ERI-1% CMC-Na for 15 and 30 sec, respectively. The bactericidal action of ERI was concentration-dependent.	Results suggest that the antibacterial activity of ERI on two types of bacteria is due to the synergistic effect of a very high negative oxidation reduction potential (−344 mV) and hydroxyl radicals (OH –). This may prove extremely useful to the prevention and treatment of periodontal diseases through daily oral care, such as rinsing the mouth out with ERI and/or brushing the teeth with ERI-1% CMC-Na.
5	Jnanadev KR., et al. 2011 [33]	China Agricultural University, Beijing, Republic of China	In vitro	To investigate the efficacy of AEW and BEW in killing *S. aureus* imbedded in biofilm and removing established *S. aureus* biofilm	*S. aureus*	Basic electrolyzed water (BEW): pH 10.8 and 11.6, acidic electrolyzed water (AEW): pH 3.5 and 2.5	AEW and BEW were generated simultaneously from the electrolysis of a 0.1% NaCl solution in a commercial EW generator (Sai Ai Environmental Protection and Technology Development Company Ltd., Guangzhou, China).	Distilled water, NAOH and HCl, BEW generated from the electrolysis of different salt solutions (BEW1, BEW2).	% of Total number of living and dead cells (absorbance at 450 nm), % of number of living cells (absorbance at 550 nm).	As the pH of BEW rose, the removal efficacy of BEW increased. BEW at pH 10.8 reduced 42% of the biofilm mass after a 2 min treatment, whereas BEW at pH 11.6 reduced 78% of the biofilm biomass. AEW1 reduced 89% biofilm viability, whereas AEW2 only reduced 13% biofilm viability. AEW at pH 2.5 and 2% HCl solution dropped biofilm viability to 5 and 4% after a 2 min treatment, respectively.	AEW could be used as a bactericide for *S. aureus* imbedded in biofilm and that BEW could be applied as a removing agent for established *S. aureus* biofilm.
6	Gomi K., et al. 2010 [34]	Tsurumi University, Yokohama, Japan	In vitro	To evaluate the disinfection effects of functional water in comparison with sodium hypochlorite solution and hydrogen peroxide solution.	Sheep prolapsed cellulose blood as inorganic substance group and saline as organic substance group, human dental pulp cells	Alkaline (pH 12.3), Strong acid (pH 2.8; 10 ppm Chlorine) and Hypochlorous (pH 6.0; 50 ppm Chlorine) electrolysis water (AEW, SAEW, or HAW)	AEW (Aoi Engineering), SAEW (Aoi Engineering), or HAW (Technomak)	Physiologic saline (PS), 3% sodium hypochlorite solution (SHS), 3% hydrogen peroxide solution (HPS).	The number of living cells (absorbance at 490 nm). CFU/mL of *E. faecalis.*	SAEW showed a weaker microbicidal activity compared to HAW in the absence of organic substance, and complete inhibition of colony-forming bacteria did not occur in the presence of organic substance. The microbicidal effect was not observed with AEW and PS.	Functional waters SAEW and HAW have good microbicidal effect in the presence of organic substance, with disinfection activity similar to that of SHS.
7	Yamada K., et al. 2010 [35]	Tokyo Dental College, 1–2-2 Masago Mihama-ku, Chiba, Japan	In vitro	To investigate the efficacy of super-oxidised water containing a high concentration of O (O-water) in destroying cariogenic and periodontopathic bacteria.	*S. sobrinus, P. gingivalis, P. intermedia, A. actinomycetemcomitans,* and *F. nucleatum.*	Super-oxidised water at low, medium or high concentration	O-water was generated in the AOE-750 (Oxy Japan Corporation, Tokyo, Japan). Three concentrations of O^−^water—low (pH 3.0), medium (pH 2.5, O^−^ concentration: ca. 3.2 x low) and high (pH 2.0, O^−^ concentration: 10x low)—were prepared.	Distilled water, hydrochloric acid solution.	CFU/mL of cariogenic and periodontophatic bacteria.	Super-oxidised water showed bactericidal activity against all cariogenic and periodontopathic bacteria tested in this study. The antibacterial effect at 37° C was higher than that at room temperature.	O-water exerts an antibacterial effect on cariogenic and periodontopathic bacteria, suggesting its potential as a disinfectant in the prevention of bacterial contamination of dental equipment
8	Ileri C., et al. 2006 [translated from Turkish] [36]	High Technology Institute, Gebze, Turkey	In vitro	To investigate the effects of EAAW on standard strains of pathogenic microorganisms at different time periods and at different concentrations.	*S. aureus* and *P. aeruginosa*	Electro-activated acidic water (EAAW) at different concentrations	EAAW was produced by direct current passed through the water by using a power source.	Sterile deionized water.	Log CFU/mL of *S.aureus* and *P. aeruginosa.*	A decrease of approximately 6.5–8.2 Log CFU/mL was detected at 2-100% concentrations of EAAW at the 10th second, depending on the microorganisms.	EAAW can be used in surface disinfection even at low (at least 2%) dilutions.
9	Gulabivala K. 2004 [22]	Seoul National University, Seoul, Korea	In vitro	To evaluate the antibacterial effect of electrolyzed tap water (Puriwater) on five major periodontopathogens cultured in vitro.	*A. actinomycetemcomitans*, *F. nucleatum*, *P. gingivalis*, *P. intermedia*, and *T. denticola.*	Puri-water: pH 8.4.	Tap water was subjected to electrolysis (30 V of DC/300 mA) for 2 min at ambient temperature using an electrolysis apparatus equipped with platinum electrodes (SciacuaTM, Puri Co., Korea).	Tap water.	CFU/mL or OD_660_	Puri-water reduced the bacterial counts to 12.6–15.4% for *A. actinomycetemcomitans, F. nucleatum, P. intermedia,* and *P. gingivalis*. Growth of *T. denticola* was not observed during 7-day incubation after exposure.	Electrolyzed tap water markedly inhibited the growth of cultured periodontopathogens.
10	Vorobjeva NV., et al. 2004 [37]	Lomonosov Moscow State University	In vitro	To evaluate the bactericidal effect of EO water on common hospital bacterial strains in vitro.	*P.aeruginosa, E. faecalis, S. aureus, E. coli, Bacillus cereus (vegetative cells and spores), Citrobacter freundii, Flavobacter* sp.*, Proteus vulgaris, Alcaligenes faecalis,* and *Aeromonas liquefaciens.*	Electrolyzed Oxidizing Water (EO):.84 ± 0.01, 1125 ± 3 mV, 43 ± 0.3 ppm Chlorine, and 4.0 ± 0.02 mS/cm	EO water was obtained from ROX-20TA electrolyzer, Hoshizaki Electric Company (Aichi, Japan) at 19.8 A and 10 V.	Deionized water.	Mean Log CFU/ mL.	The counts of the majority of the bacterial strains in the treatment samples were reduced to zero after 0.5 min of treatment, whereas the population of *B. cereus* was 3.76 log CFU/mL. After 5 min,the counts of the vegetative cells and spores of *B. cereus* were zero.	EO water renders a strong bactericidal action to both Gram-positive and Gram-negative bacteria as well as to the vegetative cells and spores of bacilli.
11	Shimada K., et al. 2000 [38]	Nihon University Japan	In vitro	To compare the bactericidal effects on cariogenic and periodontopathogenic bacteria of EW.	*S. mutans, S. sobrinus, S. mitis, S. salivarius, S. sanguis, A. biscosus, A. naeslundii, F. nucleatum, P. gingivalis, P. nigrescens, P. loeschii, P.melaninogenica,* and *A. actinomycetemcomitans.*	Acid oxidizing water (AOW), neutral oxidizing water (NtOW), acid oxidizing water with a low chlorine ppm	AOW was prepared using an Aquachid NDX- 60KMW electrolysis apparatus, (Omco O.M.C. Co., Saitama, Japan), NtOW using an Aquachid NDX- 60KH (Omco O.M.C. Co.), and AOW-LC using a Minestar 201 (Minestar Co., Tokyo, Japan).	Povidoneiodine (PI) 0.35, 0.2% chlorhexidine (CHX), Listerine (LST), 70% ethyl alcohol (Et), PBS.	CFU/mL	Bacteria incubated with NtOW, AOW, AOW-LC, LST, PI or Et did not form any colonies.	The three types of oxidizing water examined are approximately as potent at inhibiting bacterial plaque formation as conventional chemical plaque control agents.
12	Horiba N., et al. 1999 [39]	AICHI-GAKUIN University, Nagoya, Japan	In vitro	To examine Electrolized Neutral Water (ENW) bactericidial effect against bacteria isolate from infected root canals.	Methicillin-resistant *S.epidermidis*, isolated from a human nasal cavity; *B. subtilis*. 15 strains isolated and identified from infected root canals: *S. aureus, S. sanguis, L. acidophilus, S. intermedia, P. niger, P.anaerobius, V. parvula, L. rogosae, A. israelii, E. lentum, B. bifidum, P. acnes, P. endodontalis, P. melaninogenica, and F. nucleatum.*	Electrolized Neutral Water (ENW)	Ameni Clean (National Co/Matsushita Seico Co, Osaka) was used to produce ENW.	Sterilized distilled water.	CFU/mL	ENW was bactericidal against 12 strains: *S aureus, MRSE, S sanguis, P niger, Ps anaerobius, L acidophilus, L. rogosae, A israelii, E lentum, B bifidum, P. endodontalis,* and *F. nucleatum.* In addition, the ENW reduced the bacterial numbers of the other 6 strains. However, ENW showed little effectiveness against *B subtilis.*	ENW exhibits a bacteriostatic/bactericidal action against isolates obtained from infected root canals
13	Tanaka H., et al. 1996 [40]	Nagasaki University School of Medicine, Nagasaki, Japan	In vitro	To evaluate the antimicrobial activity of superoxidized water against Gram-positive and Gram-negative bacteria.	Methicillin-sensitive *S.aureus, methicillin-resistant S.aureus, S. epidermidis, S. marcescens, E. coli, P. aeruginosa,* and *B. cepacia.*	Superoxidized water: pH 2.3–2.7, ORP 1000-l 100 mV, 30 ppm chlorine.	Superoxidized water was prepared by electrolysis of tap water using the Super Oxseed alpha 1000 (Janix, Inc., Kanagawa, Japan).	0.1% chlorhexidine, 0.02% povidone iodine, 80% ethanol and sterile distilled water.	CFU/ mL	Superoxidized water reduced the viable count below the limit of detection within 10 s of contact as did 80% ethanol and 0.02% povidone iodine. Superoxidized water killed *B. cepacia* faster than 0.02% povidone iodine. The bactericidal activity of superoxidized water was superior to that of 0.1% chlorhexidine and 0.02% povidone iodine.	Superoxidized water has powerful bactericidal activity and is a low cost but powerful disinfectant.
14	Ogawa T., et al. 1998 [translated from Japanese] [42]	The Nippon Dental University, Japan	In vitro	To evaluate the bactericidal effect of soft alkaline solution water on periodontopathic bacteria.	*A. actinomycetemcomitans, P. gingivalis, P. intermedia,* and *E.coli*	Soft alkaline solution water: ORP 800 mV, pH 8–06, 60 ppm Chlorine	OXILIZER generator: OXM01 (Miura Electronics Corporation, Tokio)	Hard oxidized water: 1050 mV, pH 2.46, Chlorine concentration 30 ppm.	CFU/mL after contact time (1,5, and 10 min).	Soft alcaline water and strong acid water had a bactericidal effect against all bacteria, after 5 and 10 minutes (p < 0,05), and beyond 10 minutes (p < 0,01).	Soft alcaline water is as effective as strong acid water, having no problem with the presence of saliva. Strong acid water could be more effective in the oral environment.
15	Ogiwara K., et al. 1996 [translated from Japanese] [41]	Nippon Dental University, Japan	In vitro	To evaluate the bactericidal effect of AAW (Aqua alkalic water) to periodontopatic bacteria.	*S. aureus, E. coli, A. actinomycetemcomitans, P. gingivalis, P. intermedia,* and *F. nucleatum*	Aqua alkalic water (AAW), Aqua oxidized water (AOW),	OXILIZER water generator (OXILIZER Co., Tokyo, Japan).	Saline solution.	CFU/mL	Aa, Pg, Pi were killed in one minute (Aa; 108 to 104 CFU/ml, Pg; 108 to 103 CFU/ml, Pi; less than 108 to 103 CFU/ml) from original 100% AAW, while it took more than one minute to kill Fn, and Sa and Ec which survived after ten minutes.	The disinfectant effect of AAW could be useful for the treatment of periodontal diseases.
16	Hsieh SC., et al. 2020 [43]	Taipei Medical University, Taiwan	In vitro	To investigate the antibacterial property and cytotoxicity of EO water containing HOCl, relative to NaOCl.	Zebrafish embryo, *S. mutans and E. faecalis*	Electrolyzed Oxidizing (EO), ORP 1100 mV, 330–350 Chlorine	The ANK-Neutral Anolyte GH-40 (Envirolyte Industries International Ltd., Tallinn, Estonia) was used to produce EO water by mixing water with an over-saturated solution of sodium chloride under 110 V.	1.5% Sodium Hypochlorite, E3 medium.	CFU/mL	All the HOCl or NaOCl treatment groups showed over a 5 log 10 cfu/mL reduction in *E. faecalis* and *S. mutans* population, indicating > 99.9% antibacterial efficacy.	Both EO waters containing 0.0125 and 0.0250% HOCl revealed a remarkable but similar bactericidal effect (> 99.9%) to that of conventional NaOCl against *E. faecalis* and *S. mutans.*
17	Salisbury AM., Percival SL. 2019 [44]	5D Health Protection Group Ltd., Centre of Excellence for Biofilm Science (CEBS), Liverpool, UK	In vitro	To assess the antimicrobial and anti-biofilm efficacy of a new formulation of electrolysed water against microorganisms associated with complicated chronic wounds.	CDC Biofilm Bioreactor Model *S. aureus* and *P. aeruginosa*	Electrolysed water at concentrations of 100, 75, 50 and 25%	Water was produced by passing an electric current through deionised water, with the 2 main constituents being Sodium hypochlorite and Hypochlorous acid.	0.85% sodium chloride solution.	Mean Log_10_ densitiy CFU/mL.	No colonies of either *S. aureus* or *P. aeruginosa* were detected following antimicrobial treatment with all dilutions of the electrolysed water (p < 0.0001).	The assessment of the electrolysed water as an antimicrobial and antibiofilm agent showed exceptionally fast-acting efficacy. The use of electrolysed water as a method of controlling bioburden and biofilm in complicated chronic wounds could significantly aid wound closure.
18	Kim, S.B. 2016 [45]	Korea Institute of Industrial Technology, Ansan, Republic of Korea	In vitro	To propose a low-level hypochlorous acid solution of electrolyzed water as an alternative to mouthwash by monitoring oral bacteria though a bactericidal activity experiment	*P. gingivalls*, *P. intermedius*, *P. nigrescens*, *F. nucleatum*, *S. mutans, S. sobrinus, S. godonii, S. oralis, S. salivarius*	Low-level HOCL acid solution (electrolyzed water): pH 5–7, 3–5 mg/L Chlorine	Water was produced through a macroporous structure at Pt films electrode and an electrolysis device.	Non-treated saline solution served.	Bactericidal activity (%) for 1 min.	The low-level hypochlorous acid solution exhibited ≥99.9% bactericidal activity for all strains tested.	Low-level hypochlorous acid solution for the range of bacteria tested exhibits greater bactericidal activity for four anaerobic bacteria responsible for periodontitis and five facultative anaerobic bacteria associated with cavity development.
19	Davis JM., et al. 2007 [46]	Marquette University, Milwaukee, Wisconsin, USA	In vitro	To compare the antimicrobial action of Dermacyn, BioPure MTAD, 2% CHX (Ultradent, West Jordan, UT), and 5.25% NaOCl against *E. faecalis.*	*E. faecalis*	Super-oxidized water	Dermacyn (Oculus Innovative Sciences, Petaluma, CA, USA).	BioPure MTAD, 2% CHX, and 5.25% NaOCl, sterile distilled water.	Mean zones of microbial inhibition on aerobical and aerobical conditions.	Dermacyn and the control showed zones of microbial inhibition that were not different from each other (*p* > 0.05).	Dermacyn showed no ability to prevent the growth of *E. faecalis.*
20	Landa-Solis C., et al. 2005 [47]	Instituto Nacional de Rehabilitación, Secretarıía de Salud, Mexico, DF, Mexico	In vitro	To evaluate the disinfectant activity of Microcyn against various microbes including pathogenic vegetative bacteria in vitro.	*E. coli, S. aureus, P. aeruginosa, S. typhi,*	Super-oxidized waters (SOWs)	Microcyn (SOW) is made up by purified water which passes through anode and cathode chambers that are separated from a middle salt (NaCl) chamber by ionic membranes in a REDOX equipment (Oculus Innovative Sciences, California, USA).	Sterile, deionized water.	Log_10_ surviving Bacillus spores.	An exposure time of 30 s was enough to completely inactivate all pathogens tested in thetreatment samples (99.9999% reduction). Thus, a log10 reduction factor of 8 in the level of all pathogens occurred in the treatment samples.	Microcyn is an effective disinfectant for which sporocide activity and appropriate applications are now being validated.
21	Yoo, Y.S., et al. 2015 [49]	Dankook University, Cheonan, Republic of Korea	In vitro	To analyze and compare the antimicrobial activity of electrolyzed water using various electrodes on biofilms of oral microbes	Biofilms of oral microbes (pooled saliva of 10 healthy donors) and planktonic oral microbes. (To form biofilm of *P. gingivalis* and *T. forsythia*, the protein of *F. nucleatum* was extracted)	Electrolyzed Water using copper (EWC), silver (EWS) and platinum (EWP) electrode	Tap water was subjected to electrolysis for 5 min with 24 V of DC 400 mA using copper, silver or platinum electrode in an whole tank undivided anode chamber and cathode chamber.	Tap water.	CFU/mL, OD 590 nm and live/dead staining of *S. mutans, F. nucleatum, P. gingivalis, T. forsythia.*	Electrolyzed water using platinum electrode (EWP) exhibited antimicrobial activity against *S. mutans, P. gingivalis and T. forsythia*. The electrolyzed water using copper electrode (EWC) and silver electrode (EWS) did not affect oral microbes. EWP showed strong antimicrobial activity against the biofilm of oral microbes.	Electrolyzed water generated using a palladium electrode may have potential value as a gargle solution for prevention of oral diseases induce by pathogens and denture-related stomatitis.
22	Cho I.W., et al. 2017 [50]	College of Dentistry, Dankook University, Korea	In vitro	To investigate antimicrobial activity of recent developed EW generator for oral bacteria	*A. actinomycetemcomitans, S. mutans, F. nucleatum,* and *P. gingivalis.*	Hydrogen enriched electrolyzed water	Tap water was subjected to electrolysis for 3 min with 24 V of DC using eBio-Cleaner (ebiotech, Seoul) .	Tap water and Listerine®.	CFU/mL, LIVE/DEAD staining of cariogenic and periodontopathogenic bacteria.	eBio cleaner water showed significantly antimicrobial activity against *S. mutans* compared to tap water. The levels of *F. nucleatum*, *P. gingivalis*, and *A.a* were reduced by eBio-cleaner water. eBio-cleaner water reduced the levels of *P. gingivalis* and *A.a* by several hundred-fold compared to tap water.	The electrolyzed water generated by eBio cleaner reduced the growth of periodontopathogens and *S. mutans*. The EW generated by eBio-cleaner showed disruptive and antimicrobial effect on the salivary biofilm.
23	Lee K. 2016 [51]	Dankook University, Korea	In vitro	To investigate the antimicrobial effects of NEW on cariogenic bacteria and their biofilm, and to compare the antimicrobial activity of NEW and commercial gargle solution	*S. mutans and S. sobrinus* (To form biofilm, saliva was used).	Two types of Neutral Electrolyzed Water containing 0.05 and 0.15% sodium chloride	EW was generated from an electrolyzing distilled water containing 0.05 and 0.15% sodium chloride in an undivided anode chamber and cathode chamber.	Distilled water and commercial gargle solutions (alcohol-containing gargle for adults and a fluoride-containing gargle for children).	CFU and OD 590 nm of cariogenic bacteria.	The EW showed significant antimicrobial activity against *S. mutans and S. sobrinus.* Furthermore, the EW has more antimicrobial activity compared to the gargle solution for children. The EW and the alcohol-containing gargle significantly disrupted the biofilm. Furthermore, the count of *S. mutans and S. sobrinus* in the biofilm was decreased by both the EW and the alcohol-containing gargle. Interestingly, the EW disrupted more the biofilm and killed more the cariogenic bacteria in the biofilm than the alcohol-containing gargle.	The NEW is effective in removing cariogenic biofilm as thoroughly as the commercial gargle solutions and showed antimicrobial activity against *S. mutans* and *S. sobrinus*.
24	Gupta M., et al. 2017 [52]	Department of Microbiology, IMS, BHU, Varanasi, U.P., India	In vitro	To observe the effect of SOW in different dilutions against several pathogenic bacteria	*S. aureus, E. coli, P. aeruginosa, A. baumannii, E. faecalis,* and *K. pneumoniae.*	Superoxidised water (SOW), pH 5.0–6.5, ORP > 950 mv	SOW (Sterisol) generated by Steri-Gen® disinfectant generating system, prepared by passing the normal saline over titanium coated electrode at 9 amp	5 and 10 times dilutions of SOW.	Growth inhibition or no growth inhibition.	Undiluted SOW and 5, 10 times dilution of SOW inhibited the growth of bacteria.	Undiluted SOW prevent the occurrence of nosocomial infections. Also, the efficacy of SOW was observed in different dilutions against various microbes.
25	Lucio-Sauceda, D.G., et al. 2019 [53]	Autonomous University of Nuevo Leon, México.	In vitro	To evaluate the antimicrobial and antibiofilm activity of a novel ESS against *H. pylori.*	*H. pylori* (ATCC 700,824)	Electrolyzed solutions of superoxidation (ESS): ph 6–7, 15 ppm Chlorine, ORP 650–900 mV with	OxOral®^,^ was elaborated and supplied by Esteripharma (Mexico city, Mexico).	Tetracycline, saline solution.	Mean ± (SD) of MIC (%), The minimum bactericidal concentration (MBC value, % of biofilm inhibition).	An important antimicrobial activity of OxOral® was observed from 15 to 3.75 ppm, in those doses no difference was observed compared to 5 μg/mL tetracycline (*p* > 0.05). Therefore, 3.75 ppm of OxOral® with 99.7 ± 7.7% inhibition was established as the MIC value. The MBC value was 7.5 ppm when no visible growth was observed. However, when biofilm cells were treated with 0.938 ppm, 0.469 ppm, and 0.234 ppm of OxOral® values of 99.9 ± 5.5%, 89.1 ± 1.2%, and 35.5 ± 0.9% and of inhibition were obtained, respectively.	Antimicrobial and anti-biofilm effect of OxOral® mouthwash against *H. pylori*, and low cytotoxicity open the possibility of its therapeutic use in *H. pylori*-infected patients as adjuvant in conventional therapy.
26	Park YK., et al.; 2013 [translated from Korean] [54]	Gimcheon University, Gimcheon, Korea	In vitro	To evaluate the antibacterial effect of slightly acidic electrolyzed water (SAEW) for use as a mouth-rinse on seven oral pathogens.	*S. mutans, S. sobrinus, A. actinomycetemcomitans, P. gingivalis, P. intermedia, F. nucleatum,* and *E.coli*	Slightly acidic electrolyzed water (SAEW): pH 6.2, ORP 728–800 mV, 30 mg/L ppm chlorine	SAEW was produced in a device with hipochlorous acid (BC-360, Cosmic Round Korea Co., Seongnam, Corea)	Listerine, clorhexidine, tap water.	Mean/SD:Diameter of clear zone (mm), MIC (mg/ml or ml/ml), MBC (mg/ml or ml/ml), Bacterial strains (CFU/ml).	SAEW showed potent antimicrobial activity with a MIC value of 0.0075–0.015 mg/mL and a MBC value of 0.015–0.03 mg/mL. Mouth rinsing with SAEW showed 99.9% bacterial inhibition.	SAEW exhibited potent antimicrobial activity against all oral pathogens causing dental caries and periodontal disease.
27	Yanik K., et al. 2015 [55]	Ondokuz Mayis University, Faculty of Medicine, Kurupelit, Samsun, Turkey	In vitro	To reveal the in-vitro effect of electrolyzed water against nosocomial bacteria under different concentrations.	*A. baumannii, E. coli, E. faecalis, K. pneumoniae, P. aeruginosa, S. aureus* and eight different multidrug resistant bacteria: *A. baumannii, E. coli, vancomycin resistant Enterococcus faecium, K. pneumoniae, P. aeruginosa, methicillin resistant S. aureus, Bacillus subtilis,* and *Myroides* spp.	Electrolyzed water, pH 6.5 to 7.5, 50–700 ppm chlorine	Electrolyzed water produced by the Envirolyte® (Envirolyte® Industries International Ltd., Estonia).	Different concentrations of EW (1/1, 1/2, 1/10, 1/20, 1/50, 1/100).	No growth(−) or bacteria growth(+).	It was observed that the 1/1, 1/2, and 1/10 dilutions of electrolyzed water were effective on the standrard strains for all intervals of time.	Super-oxidized water is effective on a broad spectrum of bacteria. It was observed that Envirolyte® electrolyzed water of 100% concentration would be convenient to use for disinfection when diluted to a concentration of 1/10.
28	Vahabi S., et al. 2020 [56]	Shahid Beheshti University of Medical Sciences, Tehran, Iran	In vitro	To compare the antimicrobial effects of EW on the microorganisms of microbial plaque.	*S. salivarius, S. aureus, L. casei,* and *A. actinomycetemcomitans (JP2*).	EW was prepared at three pH values, pH 3, MBEW mildly basic electrolyzed water pH 9, and BEW-pH 11.	EW was produced using 7-plate water ionizer (iWater-sharp, Korea)	PBS,Chlorhexidine (CHX) 0.2%, CHX 0.009%, distilled water.	Mean value of CFUs and % reduction.	All EW types had a significant (*P* < 0.001) and strong antibacterial efficiency against all species in the AEW, MBEW, and BEW groups compared with the negative control. The maximum antibacterial activity of the EW at pH 3, pH 9, and pH 11 among the selected bacterial species was against *A.a* with a bacterial reduction of 100, 99.3, and 100%, respectively. The least antibacterial activity at those pH values was against *S. aureus* with a bacterial reduction of 98.04, 89.16%, and 88.75, respectively. At three EW types had an equal antibacterial potency against *L. casei* with 99.99% CFU/mL reduction. The CFU reduction of *S. salivarius* was 99.92, 99.3, and 99.94% for EW types with AEW, MBEW, and BEW, respectively.	Efficient antibacterial activities of EW at different pH values against four oral bacterial species within a 2-hour time span between water preparation and the test procedure were detected.
29	Goishi T., et al. 1996 [translated from japanese] [58]	Department of Preventive and Community Dentistry, School of Dentistry at Tokyo, The Nippon Dental University	In vitro	To investigate the bactericidal effects of AOW.	*S. mutans* and *E. coli*	Aqua Oxidizing Water (AOW), undiluted and diluted (20, 30, 40, 50%)	Water and salt was added to an electrolysis device (OXILYZER90XM-01Ltd.).	Tap water, PBS.	Number of viable bacteria CFU/mL.	Undiluted AOW was effective against the two strains tested, and the same effect was observed with 20% AOW (5-fold dilution) treatment for 30 sec.	These results suggest that 20% AOW which has bactericidal activity and is not toxic to gingival cells may be useful for dental clinical application.

*FW* Functional water, *ACC* Actual chlorine concentration, *ORP* Oxidation reduction potential, *S. mutans Streptococcus mutans*, *E. faecalis Enterococcus faecalis*, *C. albicans Candida albicans*, *P. gingivalis Porphyromonas gingivalis*, *S. sobrinus Streptococcus sobrinus*, *A. baumannii Acinetobacter baumannii*, *E. coli Escherichia coli*, *K. pneumoniae Klebsiella pneumoniae*, *P. aeruginosa Pseudomonas aeruginosa*, *S.aureus Staphylococcus aureus*, *E. faecium Enterococcus faecium*, *B. subtilis Bacillus subtilis*, *SOW* Super oxidized water, *A. actinomycetemcomitans Aggregatibacter actinomycetemcomitans*, *BEW* Basic electrolyzed water, *AEW* Acidic electrolyzed water, *SAEW* Strong acid electrolyzed water, *HAW* Hypochlorous acid water, *PS* Physiologic saline, *SHS* 3% sodium hypochlorite solution, *HPS* 3% hydrogen peroxide solution, *F. nucleatum Fusobacterium nucleatum*, *EAAW* Electro-activated acidic water, *P. intermedia Prevotella intermedia*, *T. denticola Treponema denticola*, *S. mitis Streptococcus mitis*, *S. salivarius Streptococcus salivarius*, *S.sanguis Streptococcus sanguis*, *A. viscosus Actinomyces viscosus*, *A. naeslundii Actinomyces naeslundii*, *P. nigrescens Prevotella nigrescens*, *P. loeschi*i *Prevotella loeschii*, *P.melaninogenica Prevotella melaninogenica*, *S. epidermidis Staphylococcus epidermidis*, *Bacillus subtilis B. subtillis*, *L. acidophilus Lactobacillus acidophilus*, *P. niger Peptococcus niger*, *P. anaerobius Peptostreptococcus anaerobius*, *V. parvula Veillonella parvula*, *A. israelli Actinomyces israelii*, *E. lentum Eubacterium lentum*, *B. bifidum*, *P. acnes Propionibacterium acnes*, *P. endodontalis Porphyromonas endodontalis*, *S. marcescens Serratia marcescens*, *B. cepacia Burkholderia cepacia*, *S. gordonii Streptococcus gordonii*, *S. oralis Streptococcus oralis*, *S. typhi Salmonella typhi*

**Table 3:** Electrolyzed water used against oral fungi

	Publication	Setting	Type of study	Type of question	Subject/Population	Type of water	Manufacture	Comparison	Dependant variable(s)	Main results	Authors’ conclusions
1	Okamura T., et al. 2019 [29]	Nihon University Tokyo, Japan	In vitro	To assess the antimicrobial and noxious effects of acid/alkaline electrolyzed EFWs compared with NaOCl.	*C. albicans*	Acid/alkaline-EFW. Acid: 30 ppm Chlorine; pH 2.7; ORP > 1100 mV) Alkaline: 0 ppm Chlorine; pH 11.5; ORP ≈ 800 mV	EW were provided by Miura Denshi (Nikaho, Japan)	NaOCl 6%	Mean ± SD CFU/mL and Mean ± SD viable cell number	*C. albicans* CFU reduced to 66.3% of the control after treatment with acid EW for 30 s. Treatment for longer periods yielded a time-dependent decrease in viability; no colony was present after 20 min.	Acid FW is safe and has a bactericidal effect equivalent to that of NaOCl. Because of its efficient bactericidal, and less noxious, effects on human cells, acid EFW may be a useful irrigant for effective root canal treatment.
2	Song YG., et al. 2019 [59]	Dankook University, Republic of Korea	In vitro	To determine the antifungal effect on the hyphal *C. albicans.*	*C. albicans* on resin disk	Electrolyzed water	Natural Denture Plus® (Ebioteco Co., Seoul, Korea)	Polident® and tap water	CFU/mL	The levels of *C. albicans* biofilm on the surface of the resin disk were significantly reduced with EW (p < 0.05).	The denture cleaning device showed satisfactory results for cleaning denture materials due to its antifungal activity against hyphal *C. albicans* biofilms on a denture base-resin.
3	Gunaydin M., et al.; 2014 [31]	Ondokuzmayis University, Turkey	In vitro	To investigate the in-vitro activity of superoxidized water at different concentrations against extended group of microorganisms including fungi causing hospital-acquired infections.	*Candida albicans, Candida tropicalis, Candida parapsilosis, Candida glabrata, Candida krusei, Candida lusitaniae, Trichosporon spp, Aspergillus fumigatus, Aspergillus flavus, Aspergillus niger*	Super-oxidized water: pH 6, 80 ppm chlorine at different concentrations	Medilox® (Soosan E & C, Korea) device uses salt, water and electricity and electrolyzes water.	EW at different concentrations and contact times	Absence/presence of yeast and molds	Medilox® was effective against all standard strains and all clinical isolates tested at a dilution of 1/1 and exposure time of 1 minute.	Super-oxidized water is considered as a surface disinfectant to prevent nosocomial fungal infections. Results have proved that super-oxidized water inactivates *C. krusei*, which is resistant to antifungal drugs, and *C. parapsilosis* in one minute, and at a ½ dilution.
4	Gomi K., et al. 2010 [34]	Tsurumi University, Yokohama, Japan	In vitro	To evaluate the disinfection effects of functional water in comparison with commonly used root canal irrigants such as sodium hypochlorite solution and hydrogen peroxide solution	*C. albicans* in the presence of organic substance	Electrolysis water - Alkaline: pH 12.3, Strong acid: pH 2.8; 10 ppm Chlorine, and HypochlorouS: pH 6.0; 50 ppm chlorine	AEW (Aoi Engineering), SAEW (Aoi Engineering), or HAW (Technomak)	Physiologic saline (PS), 3% sodium hypochlorite solution (SHS), 3% hydrogen peroxide solution (HPS)	CFU/mL	SAEW showed microbicidal characteristics, which were stronger than those on *C albicans* and approximately equivalent to those with HAW in the presence or absence of organic substance.	Functional water like SAEW and HAW have a good microbicidal effect under existing organic substance.
5	Yamada K., et al. 2010 [35]	Tokyo Dental College, Chiba, Japan	In vitro	To investigate the efficacy of super-oxidised water containing a high concentration of O (O-water) in destroying *C. albicans*	*C. albicans* JCM 1542	Super-oxidised water - Low: pH 3, medium: pH 2.5 or high concentration: pH 2.2,	O-water was generated in the AOE-750 (Oxy Japan Corporation, Tokyo, Japan).	Distilled water, hydrochloric acid solution (pH 2.5, 3.5 mM)	CFU/mL	O-water showed no significant fungicidal effect on *C. albicans*.	O-water showed no significant fungicidal effect on *C. albicans*.
6	Ileri C., et al. 2006 [translated from Turkish] [36]	High Technology Institute, Gebze, Turkey	In vitro	To investigate the effects of EAAS on standard strains of pathogenic microorganisms at different time periods and at different concentrations	*C. albicans* ATCC 10231	Electro-activated acidic water at different concentrations 100, 20, 10, 5, 2, 1%	EAAS was produced by direct current passed through water by using a power source. The water entering the anode and cathode area was taken from the device after being activation. Sodium chloride (NaCl, 10 g/L) was added and tap water mixture, to 2 A electric current for 15 minutes.	Sterile deionized water	Log CFU/mL	It was determined that an average population of 4.56 log CFU/mL survived for *C. albicans* at 1% dilution after 60 s.	EAAS can be used in surface disinfection even at low (at least 2%) dilutions.
7	Landa-Solis C., et al. 2005 [47]	Instituto Nacional de Rehabilitación, Mexico, DF, Mexico	In vitro	To evaluate the disinfectant activity of Microcyn against various microbes	*C. albicans*	Super-oxidized water (SOW): pH 6.2–7.8, 51–88 ppm Chlorine	Microcyn (SOW) is made by purified water which passes through anode and cathode chambers that are separated from a middle salt (NaCl) chamber by ionic membranes in a REDOX equipment (Oculus Innovative Sciences, CA, USA).	Sterile, deionized water	The surviving population of each pathogen at each sampling time was determined on TSA.	Fungicidal activity**:** 99.9% reduction after 30s exposure time.	Results indicate that Microcyn is an active fungicidal SOW.
8	Pyo K.R., et al. 2015 [60]	Dankook University, Republic of Korea	In vitro	To investigate the antifungal activity of hydrogen enriched-electrolyzed water	*C. albicans* biofilm, yeast or blastoconidia	Different electrolyzed waters using using copper, silver and palladium as electrode (EWP)	Tap water was subjected to electrolysis for 5 min with 24 V of DC 350 mA using copper, silver or palladium electrode (cylinder of 2 mm × 10 cm) in undivided chamber.	Tap water, listerine	CFU/well	In comparison with tap water, electrolyzed water showed sigficantly superior antifungal activity for *C. albicans*. The electrolyzed water and listerine exhibited fungicidal effect on *C. albicans* from 2 minutes and 4 minutes, respectively.	EWP has antifungal activity against candidal biofilm. Therefore, the EWP may be possible to use a gargle solution and a soaking solution for prevention of oral candidiasis and denture-related stomatitis
9	Gupta M., et al. 2017 [52]	Department of Microbiology, IMS, BHU, Varanasi, U.P., India	In vitro	To observe the effect of SOW in different dilutions against several pathogenic fungi	*Candida albicans, Candida krusei, Candida tropicalis, Candida parapsilosis, Aspergillus*sp. *Fusarium* sp.*, Curvularia* sp.*,* and *Bipolaris sp.*	Superoxidised water (SOW), pH 5.0–6.5, ORP > 950 mv	SOW (Sterisol) generated by Steri-Gen® disinfectant generating system. Prepared by passing the normal saline over titanium coated electrode at 9 amp.	5 and 10 times dilutions of SOW	Growth inhibition	Undiluted SOW and 5 y10 times solution of SOW inhibited the growth of *Candida* spp. Filamentous fungi was inhibited only with undiluted SOW.	Undiluted SOW prevents the occurrence of nosocomial infections specially caused of filamentous fungi.

**Table 4:** Electrolyzed water used against viruses

	Publication	Setting	Type of study	Type of question	Subject/Population	Type of water	Manufacture	Comparison	Dependant variable(s)	Main results	Authors’ conclusions
1	Morita C., et al. 2000 [61]	Osaka Medical College, Takatsuki-shi, Osaka, Japan	In vitro	To evaluate the effect of electrolyzed strong acid water with low concentration of sodium chloride on the antigenicity of the HBV surface antigen and the infectivity of HIV in vitro.	Human hepatitis B Virus surface antigen (HBsAg) purified from human plasma. HIV-1 recombinant reverse transcriptase (HIV-1 rRT).	Electrolyzed strong acid water (ESW): 1053 mV, pH 2.34, 4.20 ppm chlorine. Alkaline water: − 680 mV, pH 11.45, 0 ppm chlorine	Electrolyzed strong acid water containing sodium chloride at low concentrations was prepared in an electrolyzing apparatus (CLEANTOP WM-1, Kaigen Co. Ltd. Osaka, Japan). Ten liters of 0.05% NaCl in tap water were electrolyzed for 45 min at room temperature using a 3 A current.	HBsAg solution mixed with 300 ml of BSA solution, unelectrolyzed 0.05% NaCl solution.	Residual TCID_50_/50 μl^c^, RT Activity (OD) and residues HBs antigenicity (%).	The electrolyzed solution abolished completely HIV-1 infectivity within 20 min, in a dilution-dependent manner. When the virus particles were treated with the electrolyzed solution, RT activity was reduced in a time-dependent manner and the enzyme was completely inactivated within 5 min. The disinfection solution reduced the antigenicity of the HBsAg to below detectable levels within 2 min, in a concentration-dependent manner.	The electrolyzed solution contained only 4.2 ppm of free chlorine and showed greater efficacy against HBsAg and HIV- 1 than sodium hypochlorite.
2	Landa-Solis C., et al. 2005 [47]	Instituto Nacional de Rehabilitación, Secretarıía de Salud, Mexico, DF, Mexico	In vitro	To evaluate the disinfectant activity of Microcyn against adenoviruses and HIV-1 in vitro.	Virus films.	Super-oxidized waters (SOWs)	Microcyn (SOW) is made by purified water which passes through anode and cathode chambers that are separated from a middle salt (NaCl) chamber by ionic membranes in a REDOX equipment (Oculus Innovative Sciences, California, USA).	Sterile, deionized water.	Presence or absence of cytopathic effect on SF33 strain of HIV-1 for 5mins(TCID_50_). Presence or absence of p24 antigen. Log_10_ surviving *Bacillus* spores.	Following exposure to Microcyn, HIV-1 infectivity was not demonstrated in the viral suspension at any dilution, demonstrating complete inactivation of the HIV-1. Following exposure to Microcyn, adenovirus infectivity decreased in an inversely proportional manner to the exposure period. Taken together, these results demonstrate that virus exposure to Microcyn for 5 min achieves a log_10_ reduction factor in the viral load of 3 and that complete inactivation is achieved in 10 min of exposure to Microcyn.	Microcyn is an effective disinfectant for which sporocide activity and appropriate applications are now being validated.
3	Shimizu, Y., & Sugawara, H. 1996 [62]	Tohoku University, School of Dentistry, Japan	In vitro	To evaluate the virucidal effects of electrolyzed oxidizing water in comparison with hypochlorous acid.	HSV-1 (Miyama strain) propagated by CV-1 cells, Polio virus type 1 (Sabin strain) previously propagated with HeLa cells.	Electrolyzed oxidizing water with different Cl concentration (mg/l)	EO water was generated using JAW-035 or ND-002, manufactured by Nippon Intek Co., Ltd., by passing tap water with 0.05% NaCl as an electrolysis promoter through a narrow space between the anode and cathode, which were separated by a diaphragm.	Hypochlorous acid	Minimum concentration of Cl amount (mg/l) per condition, Minimum concentration of Cl amount (mg/l) by treatment of heat for 0, 20, and 40 mins.	A microbicidal effect was exhibited at Cl amounts of 2.74 mg/l (HSV-1) and 10.95 mg/l (polio virus) with EO water.	The results indicate that the virucidal effects of EO water differ from those of hypochlorous acid only because substances contained in EO water, such as Cl^−^, C1O_2_, H_2_O_2_, OH^+^ (hydroxyl radical) etc., seem to synergistically support such activity by balancing in a competitive state in acidic conditions.

### Electrolyzed water disinfection effectivity in the dental office setting

A total of fifteen studies have assessed the antimicrobial properties of electrolyzed water in the dental office (Table 5), using it to decontaminate dental units’ waterlines [63–67], impression materials [68–70], resin-based prostheses [33, 71, 72], titanium-based surfaces like dental implants [28], toothbrushes [22], and other surfaces or medical devices [30, 48]. Overall, studies have shown that the decontamination of dental units’ waterlines with electrolyzed water, including directly connected high-speed and low-speed hand-pieces and ultrasonic scalers [63–67], effectively inhibits bacteria and fungi growth in the long term [63–67]. Indeed, electrolyzed water showed up to 98.1% microbial killing rate for up to 6 weeks [64, 69] and similar antimicrobial activity to glutaraldehyde [33, 72]. In general terms, different kinds of electrolyzed waters have been effective in disinfecting surfaces infected with microorganisms such as *Streptococcus gordonii* [28], *Pseudomona aeruginosa* [30], *Staphylococcus spp*, *Bacillus subtilis*, and *Staphylococcus aureus* [69, 71].

**Table 5:** Electrolyzed water use in the dental office

	Publication	Setting	Type of study	Type of question	Subject/Population	Type of water	Manufacture	Comparison	Dependant variable(s)	Main results	Authors’ conclusions
1	Nakano M., et al. 2020 [translated from Japanese] [63]	Tsurumi University, Japan	CCT	To evaluate the efficacy of slightly acidic electrolyzed water. (SAEW) against the contamination of the water line of dental units	High-speed handpiece (HS-1), an ultrasonic scaler, and a cup filler of the prototype dental unit.	Slightly acidic electrolyzed water: pH 5.0–6.5	SAEW generator (PureStar, Morinaga Milk Industry). The liquid was treated in a non-diaphragmatic electrolyzer with an effective chlorine concentration of 10–80 ppm, hypochlorous acid solution.	Tap water in the same dental unit	CFU/mL of Heterotrophic bacteria	SAEW showed a significantly smaller number of heterotrophic bacteria than tap water.	SAEW continuously used for 7 years was effective for contamination control in the water line of dental units.
2	Ichioka Y., et al. 2020 [28]	Health Sciences University of Hokkaido, Japan	In vitro	To determine cytocompatibility of experimentally contaminated titanium surfaces, using a *S. gordonii* biofilm after chemical treatment with AEW or diluted H2O2 following air-abrasive debridement.	*S. gordonii* cultivated on Titanium specimens.	Alkaline electrolyzed water (AEW): chlorine concentration of 0.05%, pH 9.0	EPIOS Care® (EPIOS Corp., Tokyo, Japan).	H2O2 and NaCl	CFU/mL	The bactericidal effects of AEW and H_2_O_2_ treatments were significantly higher than that of NaCl treatment. There was no significant difference in bactericidal effect between AEW and H_2_O_2_ treatments.	AEW does not possess marked cytotoxicity and showed superior capacity in restoring titanium surface chemical properties compared with H_2_O_2_.
3	Okanda T., et al. 2019 [30]	Tokyo Medical University Hospital in Japan	In vitro	To determine the effectiveness of SAEW against biofilm-forming *P. aeruginosa* on medical devices	Seven strains of mucoid-type *P. aeruginosa* (PaM) and a non mucoid type of *P.aeruginosa* (PAO1)	Slightly acidic electrolyzed: (5,10,15,20 y 30 ppm) at 15 °C, 35 °C, and 45 °C. pH 5–6.5.	SAEW generator (Purester m-Clean; Morinaga Milk Industry Co., Tokyo, Japan) to a tap water source.	Distilled water	Presence or absence of growth of mucoid-type PaM. OD: absorbance of 580 nm.	PA cells treated with 15 ppm SAEW exhibited a partially destroyed cell wall and membrane structure and decreased cytoplasm density. Disruption of the cell structure was observed in most PA cells.	SAEW is an effective disinfectant for biofilm-forming *P. aeruginosa* and is a useful tool for disinfecting medical devices contaminated with biofilms.
4	Jeyapalan V., et al. 2018 [68]	Ragas Dental College and Hospital, Chennai, India	RCT	To comparatively evaluate the antimicrobial efficacy of three chemical disinfectants: 2.4% Glutaraldehyde (GA), 1% sodium hypochlorite (SH), and freshly prepared electrolyzed oxidizing water (EOW) on clinically derived PVS impressions	Four PVS impressions were made of the maxilla for each of the 10 subjects randomly on four different days.	EOW: 50 mg/L free chlorine, with pH of 2.5, and ORP of 1150 mv	EOW was obtained from an industrial source (Tianno Ti Anode Fabricators Pvt. Ltd., Chennai, India) using an electrolysis unit in their process with customized specifications.	2.4% GA, 1% SH, water	CFU/mL log10 count values and kill rate.	N^o^ CFU were observed in the group disinfected with EOW. EOW showed the highest log10 reduction value, which was statistically significant from both GA and SH, signifying higher an antimicrobial efficacy than the other two agents. EOW had 100% kill rate.	Freshly prepared EOW showed the highest mean log10 reduction values and 100% kill rate, indicating highest antimicrobial efficacy followed, being a promising option for disinfection of PVS impressions.
5	Fujita M., et al. 2017 [64]	Health Sciences University of Hokkaido, Japan	In vitro	To investigate the microbicidal effects of the electrolyzed water on microorganisms from DUWLs and assess any cytotoxic effects on cell lines derived from the human oral cavity	Water samples collected from Two dental units DUWLs: the air/water syringe, the high speed dental handpiece, and the cup filler	Electrolyzed water pH 7.2 ± 0.1 and ORP 793.7 n ± 9.3 mV, 21 ± 1 ppm Chlorine	Poseidon-S System (Self Medical Co., Kyoto, Japan)	Water from Dental unit reconnected to the municipal water system without a Poseidon-S	Number of CFU/mL of Gram-positive bacteria: *S.mutans, S. sanguinis, E. faecalis, A. naeslundii, M. timidum, L.casei, P. acnes*. Gram-negative Bacteria: *V. parvula, P. gingivalis, P. intermedia, F. nucleatum*.	After 18 hours of exposure to p-water, microorganisms from dental units failed to form colonies on R2A agar plates. P-water was found to reduce the viability of typical oral microbial cells from 10^8^ to 10^6^ CFU/mL, and it exhibited similar microbicidal activity against Gram-positive and Gram-negative species. This reduction represents a microbicidal rate of > 98.1%.	Poseidon-S system is an effective, additive-free disinfection system that reduces the microbial contamination of DUWLs and provides high quality water that is clean and safe for both patients and the environment.
6	Jnanadev KR., et al. 2011 [33]	V.S. Dental College, V.V. Puram, Bangalore, Karnataka, India	In vitro	To evaluate the disinfection capability of EAW as compared with 2% glutaraldehyde in disinfecting the heat cured acrylic resin prosthesis	30 acrylic specimens	Electrolyzed acid water (EAW): pH 2.3–2.4, ORP 1010–1030 mV	Two copper electrodes were placed in two beakers respectively and were connected to the positive and negative ends of a 4.5 A and6 V battery (electrolyzing apparatus). 50 mg of A grade sodium chloride (NaCl) in every 100 ml of distilled water was mixed to form 0.05% sodium chloride aqueous solution.	2% glutaraldehyde and without treatment	CFU/mL of *S. aureus.*	At 5 min, the desinfection potential didn’t show difference between EAW and 2% glutaraldehyde	The efficacy of EAW in eliminating the bacterial colonies on heat cured acrylic specimens raised from 99.88 to 99.995% as the immersion time was raised from 1 to 3 min, and complete disinfection was achieved at 5 min immersion time. As a disinfectant, EAW is as efficient as commercially available 2% glutaraldehyde at 5 min immersion time
7	Wu G., et al. 2008 [69]	Zhejiang University, Zhejiang, Republic of China	In vitro	To evaluate the feasibility of using ultrasonically nebulised electrolysed oxidising water (UNEOW) for disinfecting impressions, dental metals and gypsum casts.	1 × 1 × 0.5 cm discs of irreversible hydrocolloid impression material, gypsum cast and pure titanium	UNEOW: pH of 2.5, an ORP of 1150 mV, 50 ppm chlorine for 15, 30 and 45 min.	EOW was freshly generated using an EOW generator (SUNTECH-1000, ZH-Suntech, Zhuhai, China) and nebulised (Multisonic Compact TM, Schill Company, Probstzella Germany).	1% sodium hypochlorite for 10 min, no treatment	Kill rate (%) and log10 reduction of *Staphylococcus* spp. and spores of * Bacillus sp, S. aureus *and* B. subtilis var. niger spores.*	Immersion in EOW for 10 min resulted in a 100% kill rate for *S. aureus* and *B. subtilis* var. niger spores on titanium, alginate impressions and gypsum casts.	UNEOW used for 30–45 min with three dental materials showed satisfactory disinfection efficacy without compromising dimensional accuracy and surface quality
8	Gulabivala K., et al. 2004 [22]	Seoul National University, Seoul, Korea	In vitro	To evaluate the antibacterial effect of electrolyzed tap water (Puriwater) on toothbrushes contaminated with the periodontopathogens	*A. actinomycetemcomitans, F. nucleatum, P. gingivalis, P. intermedia,* and *T. denticola.* Seven brushes saliva collected from a total of 16 participants whom washed their mouths for 30 sec with Puri-water.	Electrolyzed water (Puri-water), pH 8.4.(exposure to EW 2 min, for 7 days).	Tap water of drinking water quality (pH 7.3, 0.76 ppm chlorine) was subjected to electrolysis (30 V of DC/300 mA) for 2 min at ambient temperature using an electrolysis apparatus equipped with platinum electrodes (280 × 120 × 90 mm, 650 g, SciacuaTM, Puri Co., Korea).	Tap water	CFU/mL or OD_660_ of periodontopathogens, aerobes, anaerobes, and streptococci bacteria.	Bacteria on toothbrushes were significantly reduced by Puri-water. *A. actinomycetemcomitans, F. nucleatum, P. intermedia,* and *P. gingivalis* remaining in the Puri-water wash ranged between 11.0 and 12.4% of the amount remaining in the tap water wash. The growth of *T. denticola* was not observed in the Puri-water wash. The number of bacteria remaining on toothbrushes after washing with Puri-water was about 50% of that after washing with tap water. Aerobes and anaerobes in saliva were significantly reduced after washing with Puri-water compared to those after washing with tap water in the same subjects (p < 0.05). Significant reduction of mutans streptococci was also observed in all participants.	Electrolyzed tap water markedly inhibited the growth of salivary bacteria as well as cultured periodontopathogens. It can be made easily in a small scale and could be useful for daily oral hygiene if used as a mouthwash and for toothbrush washing.
9	Kohno S., et al. 2004 [65]	Hiroshima University, Hiroshima, Japan	CCT	To investigate the bacterial effects of the temporary inflow of acidic electrolyzed water (AEW) on microbial contamination of the DUWL.	6 dental units: hand pieces and three-way syringes.	Acid electrolyzed water, pH 2.7, ORP 1100 mV, 32 ppm chlorine.	AEW was produced by QueenH-AP from tap water (AQUA medical Hiroshima).	Water from 3 Units that didn’t receive AEW	CFU/mL of *Legionella, Streptococcus, P. aeruginosa, E. coli, S. paucimobilis, M. mesophilicum,* and *P. stutzery*	The mean viable bacteria count was 910 ± 190 CFU/mL at the handpiece, and 521 ± 116 CFU/mL at the three-way syringe before the inflow of AEW. On the next day after the inflow of acidic electrolyzed water, bacteria were detected in only negligible amounts on the hand-piece and three-way syringe, showing significant differences from the control chair.	Acidic electrolyzed water could be as an appropiate measure against bacterial contamination of the dental unit waterline.
10	Nagamatsu Y., et al. 2001 [71]	Kyushu Dental College, Japan	In vitro	To evaluate the sterilization effects of Electrolyzed Acid Water on a resin plate.	Resin plate: (30x30x3.0 mm) heat-curing acrylic resin (UR) and a self-curing acrylic resin (QR) with and without a tissue conditioning material (TC), inmmersed in solutions with or without adding ultrasound for 1,2,5,10 mins.	Electrolyzed Strong/weak Acid Water (SW/WW): pH SW: 2.3 ± 0.3, OPR: + 1170 ± 5 mV, 50 ± 25 ppm Chlorine WW: 5.7 ± 0.2, ORP + 873 ± 5 mV, 75 ± 25 ppm Chlorine	The strong acid water was prepared by electrolyzing 0.05% sodium chloride aqueous solution with an electrolyzing apparatus (SUPER WATER mini, Hirata Corp., Osaka, Japan). The weak acid water was prepared by electrolyzing tap water with an electrolyzing apparatus (ACIDENT, J. Morita Tokyo MFG. Corp., Tokyo, Japan).	Distilled water (DW)	Number of surviving bacteria/cm^2^*: S. aureus*	The immersion treatment in the electrolyzed acid water, SW and WW, showed a marked bactericidal effect. No surviving bacteria were found on all the resin plate specimens after 1-minute treatment. No more bacteria could be detected on UR with TC after 10-minute treatment regardless of the treating method and the type of acid water.	Both the electrolyzed strong and weak acid waters are well applicable as a disinfectant for acrylic denture base showing excellent bactericidal activities in a significantly shorter treatment as compared with the conventional denture cleaning.
11	Nagamatsu Y., et al. 2016 [70]	Kyushu Dental University, Kitakyushu, Japan	In vitro	To examine the bactericidal effect of neutral water for alginate impression comparing with those of electrolyzed waters	Three Impression (EX1, EX2 and EX3) taken with an alginate and EX4 the tray.	Strong acid water (SW) pH 2.4 (0.04), ORP + 1132 (5.7), 45 ppm chlorine. Slight acid water (WW) pH 6.5 (0.27), ORP + 877 (12.2), 51 ppm Chlorine, neutral water (NW) pH 7.0 (0.07), ORP + 849 (4.5) 38 ppm Chlorine	SW was prepared by electrolyzing 0.05% sodium chloride (first-grade, Wako Pure Chemical Industries, Osaka, Japan) aqueous solution with a diaphragm by an electrolyzing apparatus (SUPER WATER mini, Hirata, Osaka, Japan). WW was prepared by electrolyzing tap water containing a specified electrolyte without a diaphragm by an automatic apparatus (ACIDENT, J. Morita Tokyo MFG, Tokyo, Japan). NW was also automatically prepared using tap water containing 5% sodium chloride. It is obtained through two steps of electrolyzation using 5% sodium chloride aqueous solution, first without and second with a diaphragm (APaqua21, Asahipretec, Kobe, Japan).	Tap water (TW)	Number of bacteria of *S. aureus*	Only 1-min treatment in these electrolyzed waters, NW and the other two types, could disinfect all the surface of the alginate impression with the tray by addition of ultrasonic cle.aning. None of the bacteria could survive on the surface of the tray after only 1-min immersion in any electrolyzed water tested, NW and the other two types. No significant bactericidal effects were found among the three electrolyzed waters tested (*p* > 0.05)	The electrolyzed waters, above all the neutral water, may be the most appropriate for the disinfection of alginate impressions.
12	Walker JT., et al. 2003 [48]	Health Protection Agency, Porton Down, Salisbury SP4 0JG, United Kingdom	In vitro	To use a model to evaluate and compare the efficacy of a variety of products based on different classes of active compound on tubing surfaces	Biofilms model: Gram-negative and oxidase-negative, planktonic bacteria	Superoxidized water 2.5% Sterilox	Sterilox, Technologies, Abingdon, United Kingdom	Sterile water, Ozone, Combizyme, Tegodor, Sporklenz, Sodium hypochlorite, Chlorhexidine, Dialox, Betadine, Parmetol, Gigasept, Grotanol, Dioxiclear, Alpron, Sanosil, Oxigenal, Bio2000, Sterilex Ultra	TVC Log_10_ CFU/cm^2^ (%) and % Reduction of Viable count/ Biofilm coverage	Sterilox resulted in a 100%reduction in the biofilm TVC and a > 95% reduction of the biofilm coverage.	The study demonstrated that while many disinfectants achieve a sufficient reduction in TVC they may not necessarily remove unwanted biofilm from the tubing surfaces as tested in this laboratory-controlled biofilm model.
13	Campregher, U.B. 2011 [72]	Universidade federal do rio grande do sul, Brasil	In vitro	To develop a low-cost portable equipment for EW production and to evaluate the microbicidal effect of EW on acrilic resin contaminated with oral microorganisms	21 denture base acrylic resin(5x5x2)	Electrolyzed Acidic Water, pH 3.0 and oxidation reduction potential (ORP) of 1150 mV	EAW was produced through an electrolysis process of an aqueous NaCl solution (5%) in an electrolytic two chambers cell with a separation membrane between the chambers (30 V, 2 A).	Distilled water, Glutaraldehyde 2%	Turbidity %	Specimens treated with EAW didnt provoke turbidity on the culture media	EAW was effective in the disinfection of the acrylic resin contaminated by immersion of it for 10 minutes
14	Mishima S., et al. 2016 [66]	Kyoto University Hospital, Japan	RCT	To evaluate the effectiveness of inhibition of bacterial proliferation using the purification system to supply neutral electrolytic water for refining the waterworks in DUWLs.	Water samples were collected 6 dental units from the high-speed handpiece, the three-way syringe and the gargle water.	Neutral Electrolytic Water, pH of 6.5–7.5, 5 ppm chlorine	Poseidon S (Self Medical Co., Osaka, Japan) is purified electrolytic water from the tap water.	Tap water	CFU/mL pathogenic bacterial species	Before cleaning of DUWLs, the number of heterotrophic bacterium discharged from high-speed handpiece was 2.3 × 10^5^ cfu/mL, low-speed handpiece was 3.2 × 10^4^ cfu/mL, and threeway syringe was 1.3 × 10^5^ cfu/mL. After 3 and 14 months, the number of heterotrophic bacteria discharged from the high-speed handpieces, three-way syringes and gargle water was < 30 cfu/mL in EW treated group.	The water purification system using neutral electrolytic water was effective to control the proliferation of bacteria and could maintain a hygienic environment in DUWLs.
15	Martin, M., Gallagher, M. 2005 [67]	Liverpool Dental Hospital, UK	RCT	To determine the efficacy of super-oxidised water (Optident/Sterilox) in the decontamination of DUWLs.	Ten Adec Cascade units were used in the trial with independent reservoirs (The turbine, slow-speed, three-in-one syringe and cup-filler outlets).	Super–oxidised water	Optident/Sterilox,Optident (Ilkley, Yorkshire). On demand, the saline solution was passed through the generator producing concentrated super-oxidised water.	Not declared	Total CFU recover (range) of *oral streptococci, Actinomyces spp, oral anaerobes, Enterobacteria, Pseudomonads, Candida, Legionella,* and *Mycobacterium* spp.	The results show the number of CFU recovered from the first two weeks of the trial; after this period no further bacteria were recovered. After 7 d no further bacteria were recovered from the units.	Super-oxidised water was successful in the removal of bacteria from dental unit water supplies. Complete removal required the treatment with a purge phase of concentrated disinfectant and a maintenance phase of at least two weeks.

*S. gordonii Streptococcus gordonii*, *P. aeruginosa Pseudomonas aeruginosa*, *H2O2* Hydrogen peroxide, *Streptococcus mutans S.mutans, Streptococcus sanguinis S. sanguinis*, *Enterococcus faecalis E. faecalis*, *A. naeslundii Actinomyces naeslundii*, *M. timidum Mogibacterium timidum*, *L. casei Lactobacillus casei*, *P. acnes Propionibacterim acnes*, *V. parvula Veillonella parvula*, *P. gingivalis Porphyromonas gingivalis*, *P. intermedia Prevotella intermedia*, *F. nucleatum Fusobacterium nucleatum, DUWL* Dental unit water line *S. aureus Staphylococcus aureus, B. subtilis Bacillus subtilis*, *A.actinomycetemcomitans Aggregatibacter actinomycetemcomitans*, *T. denticola Treponema denticola*, *E. coli Escherichia coli*, *S. paucimobilis Sphingomonas paucimobilis*, *M. mesophilicum Methylobacterium mesophilicum*, *P. stutzery Pseudomonas stutzery*

### Compatibility of electrolyzed waters for dental materials

Some studies tested the disinfection potential and material compatibility of electrolyzed water with dental materials [33, 68, 70–72]. For example, electrolyzed water effectively disinfected impressions made from polyvinyl siloxane [68] and alginate [69, 70] without compromising their dimensional integrity or surface accuracy. Apart from that, the antimicrobial use of electrolyzed water was also demonstrated to be harmless to acrylic resin dentures contaminated with bacteria such as *S. aureus* [33, 71, 72].

### Risk of bias assessment

The RoB and quality of reporting assessment using the NPQIP tool led to a total of 756 entries (Additional file 3). Of them, only 35.18% were answered as ‘Yes’, while 48.15% as ‘No’ and the rest were marked as ‘NA’ (Not applicable). The tool sections ‘Animal information’ and ‘Reagents (in vivo)’ did not apply to the included studies.

All of the studies scored ‘Yes’ in items #1 and #2 regarding sample size and collection specification. Items #3 and #4 regarding experiment replications and the use of uncommon statistical tests showed similar results with 52.46 and 45.90% of positive answers, respectively. Only one study was rated as ‘Yes’ in item #5, regarding the specification of using one or two-sided t or z tests. Most articles answered items #6, #7, and #8, about using *P* values and estimates as medians or averages, as ‘Yes’, being 59.02, 68.85, and 55.73%, respectively. However, items #9-#12 (62.29, 75.40, 63.93, and 50%) mainly were answered as ‘No’ due to articles not reporting the definition of error bars, not assessing data normality, not calculating variation within groups and not analyzing the variance. In addition, most articles did not specify how the sample size was obtained, resulting in items #13 and #14 with 85.24 and 81.96% of ‘No’ answers. In this sense, items #15, #16, and #17, regarding exclusions, items #18 and #19, regarding randomization, and items #20 and #21, regarding blinding, were mostly (> 80%) answered as ‘No’. Apart from that, most articles specified if their antibodies were profiled and the cell lines used, being items #22 and #23 answered as ‘Yes’ in 83.61 and 65.57%, respectively. Although, no articles specified if the cell lines were authenticated or if they were tested for mycoplasma contamination in items #24 and #25. Finally, most articles answered ‘Not applicable’ for the ‘Human studies’ items #26–30. In this sense, the few human studies included did identify a committee approving the protocol and the use of informed consent, but did not have clinical trial registration numbers nor used the CONSORT checklist.

## Discussion

Electrolyzed water is obtained from the combination of tap water with a sodium chloride-derived solution passed through an electrolysis chamber, and it has been proven to be a cheap and environment-friendly alternative to disinfect different types of contaminated surfaces and inert materials due to its vast microbicide properties [53]. In the present revision, the included studies assessed several types of differently obtained electrolyzed waters, which showed a high disinfection potential when used to deal with different oral conditions, including caries, periodontal disease, and root canal system infection. These beneficial effects were achieved by reducing the amount of CFUs of disease-specific bacteria, which was higher than tap water or saline solution, reaching a disinfection potential similar to that described for chlorhexidine or sodium hypochlorite solution.

Different antimicrobial agents have been tested to reduce the cross-infection potential during dental procedures, but many of them are not completely innocuous for dental materials or safe for oral tissues [68]. In the herein included studies, electrolyzed water was demonstrated to have a broad antimicrobial spectrum and to be highly efficient in the dental office disinfection against viruses, fungi, and bacteria, being also compatible with most dental impression materials, gypsum, resin-derived materials, and dental implants. Moreover, different kinds of electrolyzed waters have been put into dental unit water lines connected to high-speed and low-speed hand-pieces, ultrasonic cavitrons, and air sprays, showing an effective capacity to control microorganisms and being harmless to oral tissues. In fact, six of the included studies showed that electrolyzed water does not produce adverse effects in patients when used as a daily mouth rinse, nor produce cytotoxic effects on cultured human bone marrow-derived mesenchymal stem cells [17–21, 28].

These critical findings must be considered in the context of the COVID-19 pandemic since dentistry has become one of the health fields with the most significant impact on its transmissibility [73]. Indeed, the main route of airborne transmission and the consequent possible viral dissemination during dental practice originated mainly through the aerosolization produced by high-speed hand-pieces and ultrasonic scalers [63–67]. In this context, electrolyzed water has also shown to have great microbicidal potential against viruses that are easily transmitted during dental care, such as the human hepatitis B virus, human immunodeficiency virus, herpes simplex virus type 1, and poliovirus type 1, in a time-dependent manner [47, 61, 62]. In addition, the effectivity of electrolyzed water against the SARS-CoV-2 virus has also been tested in vitro, showing promising results without showing cytotoxicity [32]. Indeed, the SARS-CoV-2 virus exposed to undiluted electrolyzed water for 60 seconds significantly reduced its infectivity and replication [32]. Furthermore, oral and nasal rinse with electrolyzed water has also been shown to be protective against COVID-19 infection in a clinical trial [74]. In this study, only 1.2% of the study group that rinsed with electrolyzed water reported COVID-19 incidence, while 12.7% of the control group that did not rinse with electrolyzed water became infected [74]. The effectivity of electrolyzed water has also been proved against enveloped and non-enveloped viruses by breaking chemical bonds and changing surface proteins, mainly through its redox potential, compromising the viral envelope, viral enzymes, and viral nucleic acids [61]. Therefore, among other viruses, electrolyzed water could protect against SARS-CoV-2 infection and contamination in the dental office.

Among the different electrolyzed waters, the most frequently assessed were ‘electrolyzed hydrogen water’, ‘aqua oxidizing water’, ‘acid water’, ‘superoxidized water’, and ‘oxidative potential water’, with subtle variations of chlorine concentration and pH among them [17–27]. However, some cases, including ‘strong acid water’ and ‘weak acid water’, could corrode metal surfaces and instruments when prepared with high sodium chloride concentrations [61]. Moreover, electrolysis is capable of sterilizing tap water and reactivating the free chlorine in it, making it able to disinfect surfaces and water deposits against both oral and nosocomial infection-causing bacteria, without evidence of corrosion [75].

In this context, mouthwashes are commonly used as a daily antiseptic complement to toothbrushing for the maintenance of oral hygiene; however, there is still no clear evidence of their long-term effects on the health-compatible oral microbiota [19, 21]. In this sense, electrolyzed water has also been proven to be safe and significantly inhibit the growth of *Streptococci* in saliva, compared with saliva from patients that rinsed with tap water [76]. Indeed, the common active components of electrolyzed water, independent of their varying pH, chlorine, hypochlorous acid, and hypochlorous acidic ion, are thought to be responsible for electrolyzed water’s antibacterial capacities [29].

On the other hand, fungicides are not commonly used for disinfecting the dental office, but when used, they tend to leave residues and are not entirely environmentally friendly. Likewise, in the analyzed studies, the antifungal activity of electrolyzed water was evaluated as an alternative. Nine studies showed that electrolyzed effectively inhibited the growth of *C. albicans* [29, 31, 34–36, 47, 52, 59, 60]. In this context, electrolyzed water antifungal activity relies mainly on OH, which causes structural damage to cell walls, leading to the leakage of K^+^ and Mg^+ 2^ ions and consequently compromising fungi cell function. Besides, its effectivity may also depend on its available chlorine concentrations and the thickness of the fungi cell wall [77]. Even so, other disinfectants, such as sodium hypochlorite, are more time-efficient than electrolyzed water [29].

One of the limitations of our study was the substantial methodological heterogeneity found among the included studies, which impeded the adequate pooling of results and the performance of a meta-analysis. The most common sources of heterogeneity were the different ways to prepare electrolyzed water, the diversity of the assessed pathogens, and the different exposure times. In addition, sample sizes and the adequacy of their calculation were not considered during data extraction and drawing conclusions. Indeed, normality of data distribution, sample size calculation, and error bars definition were frequently unreported. Moreover, most of the included studies had an in vitro design, which rarely reported the randomization of interventions or the blinded assessment of results, thus limiting precise clinical conclusions. However, our vast search strategy and identification of articles written in languages different from English make our study publication bias low; therefore, probably capturing most of the state-of-the-art literature regarding the use of electrolyzed water for microbial control in the dental setting. Besides, our assessment of the risk of bias among the studies suggests a cautious interpretation of the included study results.

Different concentrations of free chlorine, ranging from 4 to 88 ppm, were used for fabricating electrolyzed waters for diverse applications, being 50 ppm the most used concentration [17–27]. For the disinfection of the dental office and its appliances, the most effective electrolyzed waters were ‘Poseidon-S’ – pH 7.2 ± 0.1, ORP 793.7 ± 9.3 mV, chlorine 21 ± 1 ppm – [64] and ‘Ultrasonically nebulized electrolyzed oxidizing water’ – pH 2.5, ORP 1150 mV, chlorine 50 ppm – [69]. Moreover, regarding its direct use as a mouthwash, ‘Electrolyzed hydrogen water’ – ORP − 600 mV ~ − 700 mV, hydrogen 1.5 ppm –[17] and ‘Acid water’ – pH < 2.7, ORP > 1100 mV – [21] were the most effective in reducing caries associated bacteria and controing gingivitis, respectively. However, special storage and maintenance conditions are needed to ensure microbicidal properties. In fact, storage over 40 days and daylight exposure might affect electrolyzed water disinfecting potential or even inactivate it [33, 55]. Regardless of these suggestions, it is imperative to carry out new and more extensive clinical trials in different scenarios to ratify the electrolyzed water clinical implementation. However, substantial evidence points out that its use for disinfecting the whole dental office equipment and materials is effective, eco-friendly, and most important, safe.

## Conclusion

The antimicrobial properties of electrolyzed water are diverse and their effective ability to disinfect contaminated surfaces, dental materials, and equipment make their use recommendable in the SARS-CoV-2 pandemic dental setting.

## Supplementary Information

**Additional file 1: Supplementary Material 1.** Search Strategies carried out in MEDLINE via Pubmed, EMBASE, Scopus, Web of Science, Cochrane’s CENTRAL, and LILACS databases.

**Additional file 2: Supplementary Material 2.** Excluded articles and their respective reasons for exclusion.

**Additional file 3: Supplementary Material 3.** Risk of Bias assessment using the NPQIP tool.

## Data Availability

All data generated or analyzed during this study are included in this published article.
