# Supplementary material for: Electrolyzed water for the microbiologic control in the pandemic dental setting: a systematic review
Source: BMC Oral Health. 2022 Dec 9;22:579. doi: 10.1186/s12903-022-02528-0 (PMC9733258; doi:10.1186/s12903-022-02528-0)
Supplement: Supplementary file 1 — Additional file 1: Supplementary Material 1. Search Strategies carried out in MEDLINE via Pubmed, EMBASE, Scopus, Web of Science, Cochrane’s CENTRAL, and LILACS databases. [file 12903_2022_2528_MOESM1_ESM.docx]

**Supplementary Material 1**. Search Strategies carried out in MEDLINE via Pubmed, EMBASE, Scopus, Web of Science, Cochrane’s CENTRAL, and LILACS databases were the following:

**MEDLINE via Pubmed**

172 hits

(((((((((((electrolyzed strong water) OR (electrolyzed neutral water)) OR (electrolyzed oxidizing water)) OR (acid electrolyzed water)) OR (acidic electrolyzed water)) OR (high oxidation potential water)) OR (aqua oxidizing water)) OR (oxidative potential water))) OR ((electrolyzed water OR "superoxidized water" [Supplementary Concept]) OR (superoxidized water) OR (electrolyzed weak acid water) OR (electrolyzed strong acid water) OR (sterilox) OR (neutral electrolyzed water) OR (electrolyzed acid water) OR (electrolysed water))) AND ((((((((((((agents, antiviral[MeSH Terms]) OR (antiviral agent) OR (antiviral drug) OR (antiviric) OR (antiviridal) OR (inactivation, viral[MeSH Terms]) OR (viral inactivation) OR (virus inactivation) OR (virus) OR (virion)) OR (virucidal)) OR (virucidal effect)) OR (bactericidal)) OR (oral bacteria)) OR (bactericidal effect)) OR (bactericidal action)) OR (bacteriological study)) OR (bacteriological action)) OR (antibiotic)) OR (antibiotic effect))) AND ((((((((("Dentistry"[Mesh]) OR (dentistry) OR (odontology) OR (periodont*) OR (endodont*) OR (oral surgery) OR (dental estethics) OR (oral medicine) OR (oral surgical procedure) OR (orthodont*) OR (oral pathology) OR (prosthodont*)) OR (clinical application)) OR (dental field)) OR (clinical dentistry)) OR (dental region)) OR (plaque formation)) OR (denture cleanser)) OR (infection control))

**EMBASE**

9 hits

#1 = 'electrolyzed water' OR 'electrolyzed reduced water' OR 'electrolyzed neutral water' OR 'electrolyzed oxidizing water' OR 'acid electrolyzed water' OR 'acidic electrolyzed water' OR 'high oxidation potential water' OR 'aqua oxidizing water' OR 'oxidative potential water' OR 'superoxidized water' OR 'electrolyzed weak acid water' OR 'electrolyzed strong water' OR 'sterilox' OR 'neutral electrolyzed water' OR 'electrolyzed acid water' OR 'electrolysed water'

#2 = 'antivirus agent' OR 'antiviric' OR 'viral inactivation' OR 'virus inactivation' OR 'virus' OR 'virion' OR 'virucidal activity' OR 'bactericide' OR 'bactericidal activity' OR 'bacteriological study' OR 'bacteriological action' OR 'antibiotic agent' OR 'antibiotic effect' OR 'mouth flora'

#3 = 'dentistry' OR 'dentistry'/exp OR dentistry OR 'operative dentistry'/exp OR 'operative dentistry' OR 'periodontics' OR 'periodontics'/exp OR periodontics OR 'endodontics' OR 'endodontics'/exp OR endodontics OR 'stomatology' OR 'stomatology'/exp OR stomatology OR 'orthodontics' OR 'orthodontics'/exp OR orthodontics OR 'prosthodontics' OR 'prosthodontics'/exp OR prosthodontics OR 'oral pathology' OR 'odontology' OR 'odontology'/exp OR odontology OR 'oral surgery'/exp OR 'oral surgery' OR 'tooth disease'/exp OR 'tooth disease' OR 'dental field' OR 'dental region' OR 'clinical dentistry' OR 'denture cleaner'/exp OR 'denture cleaner'

#4 = #1 AND #2 AND #3

**Scopus**

97 hits

( TITLE-ABS-KEY ( "electrolyzed water"  OR  "electrolysed water"  OR  "superoxidized water"  OR  "electrolyzed weak acid water"  OR  "electrolyzed strong acid water"  OR  "neutral electrolyzed water"  OR  "electrolyzed acid water"  OR  "sterilox"  OR  "electrolyzed strong water"  OR  "electrolyzed neutral water"  OR  "electrolyzed oxidizing water"  OR  "acid electrolyzed water"  OR  "acidic electrolyzed water"  OR  "high oxidation potential water"  OR  "aqua oxidizing water"  OR  "oxidative potential water" ) )  AND  ( ALL ( "antiviral agent"  OR  "antiviral drug"  OR  "antiviric"  OR  "viral inactivation"  OR  "virus inactivation"  OR  "virus"  OR  "virion"  OR  "virucidal"  OR  "virucidal effect"  OR  "bactericidal"  OR  "oral bacteria"  OR  "bactericidal effect"  OR  "bactericidal action"  OR  "bacteriological study"  OR  "bacteriological action"  OR  "antibiotic"  OR  "antibiotic effect" ) )  AND  ( ALL ( "dentistry"  OR  "odontology"  OR  "stomatology"  OR  "periodontics"  OR  "periodontal"  OR  "periodontology"  OR  "endodontics"  OR  "endodontology"  OR  "root canal"  OR  "oral surgery"  OR  "dental estethics"  OR  "oral medicine"  OR  "oral surgical procedure"  OR  "orthodontics"  OR  "orthodontic"  OR  "oral pathology"  OR  "prosthodontics"  OR  "prosthetics"  OR  "prosthesis"  OR  "dental field"  OR  "clinical dentistry"  OR  "dental region"  OR  "plaque formation"  OR  "denture cleaner" ) )

**Web of Science**

6 hits

ALL=(( "electrolyzed water" OR "electrolysed water" OR "superoxidized water" OR "electrolyzed weak acid water" OR "electrolyzed strong acid water" OR "neutral electrolyzed water" OR "electrolyzed acid water" OR "sterilox" OR "electrolyzed strong water" OR "electrolyzed neutral water" OR "electrolyzed oxidizing water" OR "acid electrolyzed water" OR "acidic electrolyzed water" OR "high oxidation potential water" OR "aqua oxidizing water" OR "oxidative potential water") AND ("antiviral agent" OR "antiviral drug" OR "antiviric" OR "viral inactivation" OR "virus inactivation" OR "virus" OR "virion" OR "virucidal" OR "virucidal effect" OR "bactericidal" OR "oral bacteria" OR "bactericidal effect" OR "bactericidal action" OR "bacteriological study" OR "bacteriological action" OR "antibiotic" OR "antibiotic effect") AND ( "dentistry" OR "odontology" OR "stomatology" OR "periodontics" OR "periodontal" OR "periodontology" OR "endodontics" OR "endodontology" OR "root canal" OR "oral surgery" OR "dental estethics" OR "oral medicine" OR "oral surgical procedure" OR "orthodontics" OR "orthodontic" OR "oral pathology" OR "prosthodontics" OR "prosthetics" OR "prosthesis" OR "dental field" OR "clinical dentistry" OR "dental region" OR "plaque formation" OR "denture cleanser"))

**Cochrane’s CENTRAL**

6 hits

( "electrolyzed water" OR "electrolysed water" OR "super oxidized water" OR "electrolyzed weak acid water" OR "electrolyzed strong acid water" OR "neutral electrolyzed water" OR "electrolyzed acid water" OR "sterilox" ) AND ( "dentistry" OR "odontology" OR "stomatology" OR "periodontics" OR "periodontal" OR "periodontology" OR "endodontics" OR "endodontology" OR "root canal" OR "oral surgery" OR "dental estethics" OR "oral medicine" OR "oral surgical procedure" OR "orthodontics" OR "orthodontic" OR "oral pathology" OR "prosthodontics" OR "prosthetics" OR "prosthesis" ) in All Text - (Word variations have been searched)

**LILACS**

9 hits

eletrolisada OR electrolizada OR electrolyzed OR superoxidada OR superoxidized OR sterilox [Palabras]
